# Triplet‐Singlet Emission of d‐Block Metal Complexes Characterized by Spin‐Orbit Natural Transition Orbitals

**DOI:** 10.1002/open.202300291

**Published:** 2024-03-05

**Authors:** A. Zaichenko, J. Autschbach

**Affiliations:** ^1^ Department of Chemistry University at Buffalo State University of New York Buffalo NY 14260-3000 USA

## Abstract

Spin‐orbit natural transition orbital (SO‐NTO) methodology, recently developed in our group for complete and restricted active space (CAS/RAS) wavefunction calculations, is applied to analyze triplet‐to‐singlet emission in transition metal complexes. The lowest‐energy (longest‐wavelength) spin‐forbidden transition $T_{1}\to S_{0}$ is studied for for [Ir(pbt)2(acac)] and [Re(CO)4(pbt)] and the complexes [W(CO)4(bpy)] and [Mo(CO)4(bpy)]. For the latter complexes, spin‐forbidden transitions from higher spin‐triplet levels are additionally analyzed. SO‐NTOs are compared with spin‐free NTOs for the transitions under consideration. The major assignment of a spin‐forbidden transition is obtained from the spin‐free NTO analysis, while the source of intensity of the electronic transition is revealed by the SO‐NTOs.

## Graphical abstract

In the present study, the SO‐ NTO technique was applied to analyze triplet‐to‐singlet emission in a set of transition metal complexes with Mo, W, Re, and Ir. The source of intensity in the spin‐orbit forbidden transitions has been demonstrated and discussed.

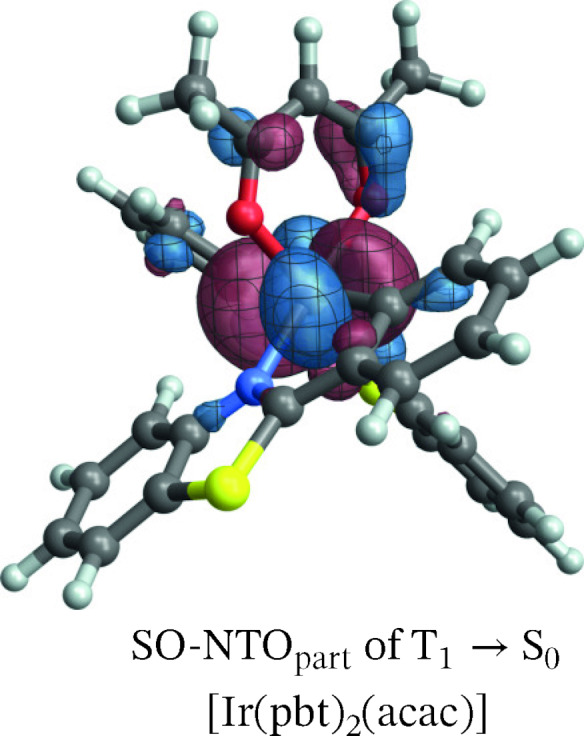

## Introduction

The theoretical study of transitions between electronic states with different spin multiplicity is of great interest for a comprehensive analysis and understanding of light‐emitting processes in transition metal complexes, among other types of systems. Specifically, the study of radiative transitions in metal complexes has for many decades been important not only for technical applications but also in the development of fundamental photochemical concepts.[^1^, ^2^, ^3^, ^4^, ^5^, ^6^, ^7^]

A large number of applications of phosphorescent materials centers on the emission from the lowest triplet state (T_1_) of systems possessing a closed‐shell singlet ground state (S_0_). The spin selection rule renders the intensity of electronic triplet‐singlet transitions between pure spin states as strictly forbidden. However, the spin selection rule can be weakened or rendered altogether ineffective in the presence of the spin‐orbit (SO) interaction and the associated SO coupling (SOC) of the electronic states. In the presence of SOC, the electron spin is no longer ‘a good quantum number’, and formally spin‐forbidden transitions may acquire intensity due to the mixing of states of different spin multiplicity.^[8]^ SOC is therefore a key ingredient for the theoretical analysis of triplet‐singlet emission.

Natural Transition Orbitals (NTOs) represent a well‐established and powerful tool for the analysis of electronic transitions.^[9]^ NTOs provide a compact and transparent representation of electronic transitions, specifically, how and to which degree two different electronic states are connected to each other via single‐particle transitions. This is accomplished by singular value decomposition of the transition density matrix expressed in some one‐particle basis such as the set of molecular orbitals (MOs) or the atomic orbitals (AOs) chosen as the basis set for a calculation. NTOs have been applied extensively to spin‐allowed transitions. NTOs and related concepts can also be used for assigning spin‐forbidden transitions,[^10^, ^11^, ^12^] although initial applications did not specifically consider the components of the transition that generate the intensity. For the analysis of transition moments specifically for spin‐forbidden transitions, spin‐orbit natural transition orbitals (SO‐NTOs) were introduced by our group recently^[13]^ and implemented in the OpenMolcas code.[^14^, ^15^] The SO‐NTOs are generated from the one‐particle transition density matrix for a pair of SO states and provide the crucial information about non‐vanishing components in the spin‐mixed states that are directly responsible for the intensity. So far, only a proof of concept was demonstrated.^[13]^ This paper constitutes the first part of a planned series of applications of SO‐NTOs to analyze spin‐forbidden transitions in complexes with transition metals or rare earths.

The present study demonstrates the application of SO‐NTOs to investigate radiative emission in transition metal (d‐block element) complexes based on multi‐configuration (MC) restricted active space self‐consistent field (RASSCF) and complete active space self‐consistent field (CASSCF) wavefunction theory (WFT) calculations. Dynamic correlation has been included by multi‐state multiconfiguration pair density functional theory (MC‐pDFT) or second‐order perturbation theory (PT2), as detailed later. The complexes listed in Table 1 were selected for the study. Complexes **3** and **4** containing W and Mo, respectively, were recently reported^[6]^ as of high interest regarding their emission. Complexes **1** and **2** containing Ir and Re, respectively, were studied previously with two‐component relativistic time dependent density functional theory (TD‐DFT).^[5]^ Good agreement with experimental emission lifetimes and the zero‐field splitting was obtained with the TD‐DFT approach, and therefore these systems serve as useful benchmarks for the present investigation by CAS/RAS‐type approaches.

**Table 1:** Metal complexes studied in this work^[a]^ and numbering used in the text.

Complex	Numbering
[Ir(pbt)_2_(acac)]	**1**
[Re(CO)_4_(pbt)]	**2**
[W(CO)_4_(bpy)]	**3**
[Mo(CO)_4_(bpy)]	**4**

[a] pbt=2‐phenylbenzothiazolate. acac=acetylacetonate. bpy=2,2’‐bipyrimidine.

Photo‐physical data are reported in this work for the first triplet‐singlet transitions for all four complexes, and for a second (potential) emission transition for complexes **3** and **4**, based on complete and restricted active‐space wavefunction calculations, in some cases augmented by a treatment of the dynamic correlation for the state energies. SO‐NTOs are presented and compared with active space orbitals and spin‐free NTOs to analyze the source of intensity of the spin‐forbidden transitions. The study illustrates applications of SO‐NTOs, which may be useful to optimize the emission characteristics of metal complexes.

## Theoretical and Computational Details

Optimizations of the molecular structures (‘geometries’) for ground and excited states were performed with the Gaussian 16 (G16)^[16]^ program package using the Becke three‐parameter exchange and Lee‐Yang‐Parr correlation (B3LYP)[^17^, ^18^, ^19^, ^20^] functional. For the optimizations, relativistic small core (RSC) effective core potentials with matching valence basis sets (ECPXXMWB with XX=60 for W, Re, Ir and XX=28 for Mo) were used for the central metal atom.^[21]^ For Ir, g‐functions (ℓ=4 ) were removed from the basis because of the rather large size of the complex (**1**). For the ligand atoms H, C, N, O, S the def2‐SVP basis sets^[22]^ were used. Optimizations of triplet state geometries were performed with TD‐DFT response calculations with and without invoking the Tamm‐Dancoff approximation (TDA), solving for the 10 lowest triplet states with optimization of corresponding emitting states as targets for all systems except **1**. For the latter, a spin‐unrestricted B3LYP structure optimization (UB3LYP) was carried out for the lowest triplet state (T_1_). Optimized structures for **3** and **4** were virtually identical with or without invoking the TDA. For **2**, small differences in the metal‐ligand angles were found. The TDA structures were retained for further calculations [see Suppplementary Information (SI), Figures S1–S5 and Tables S2–S11]. The B3LYP functional was selected for consistency with structures and state assignments from previous TD‐DFT studies of the complexes.[^5^, ^6^]

All multi‐configurational and multi‐reference calculations were performed with an up‐to‐date ‘master’ branch of the Open‐Molcas program,[^14^, ^23^] employing restricted and complete active‐space self‐consistent field (RASSCF, CASSCF) methods.[^24^, ^25^] These calculations were augmented with multi‐configurational pair‐density functional theory (MC‐pDFT)[^26^, ^27^] or extended multi‐state restricted active‐space perturbation theory to second order (XMS‐RASPT2, abbreviated as PT2 in the following)[^28^, ^29^] to treat the dynamic correlation. Calculations were performed in an all‐electron fashion, using the exact two‐component (X2C) relativistic Hamiltonian^[30]^ as implemented in OpenMolcas. The SO interaction was treated by restricted active space state interaction (RASSI)[^31^, ^32^, ^33^, ^34^] of pure spin states. The SO Hamiltonian was constructed based on the atomic mean‐field integral (AMFI) approach^[35]^ implemented in OpenMolcas. Electron repulsion integrals were computed using the resolution‐of‐identity Cholesky decomposition approximation (RICD).^[36]^ The ‘translated’ Perdew‐Burke‐Ernzerhof (PBE)[^37^, ^38^] functional was used in the MC‐pDFT steps. Atomic natural orbital relativistic ANO‐RCC‐VTZP^[39]^ basis sets were used for the metal centers. ANO‐RCC‐VDZP^[40]^ and the minimal basis contraction ANO‐RCC‐MB^[40]^ were used for non‐hydrogen and hydrogen ligand atoms, respectively. Point group symmetry was not explicitly used in the wavefunction calculations; however, for the large active spaces needed for **3** and **4**, ‘supersymmetry’ designations were used to prevent orbital mixing akin to the *C*_2*v*_ point group to improve convergence and reduce computation times. Active spaces were selected and assigned with consideration of the physical nature of the considered systems, the electronic transitions, and computational performance, as detailed below.

The NTOs, SO‐NTOs and transition probabilities were computed with the RASSI module of OpenMolcas. The main contributions to the spin‐free transitions were obtained from the RASSCF calculations. For **1**, which has more strongly mixed SO states and the smallest active space, 30 singlet and 30 triplet states were coupled in RASSI for the transitions analysis, similar to the previous proof‐of‐concept calculations for the complex [Ir(ppy)_3_] (ppy=2‐phenylpyridine).^[13]^ The other systems required larger active spaces but evidenced weaker SO‐induced mixing of different spin states. Accordingly, 10 singlet and 10 triplet spin‐free (SF) states were used for the RASSI step. CAS‐CI calculations (i. e., without further orbital optimization) for **3** and **4** including 30 states each for spin singlet and triplet multiplicities demonstrated that there is no significant contribution of higher‐energy SF states for the SO states of T_1_ to T_3_ parentage.

**Figure 1:**
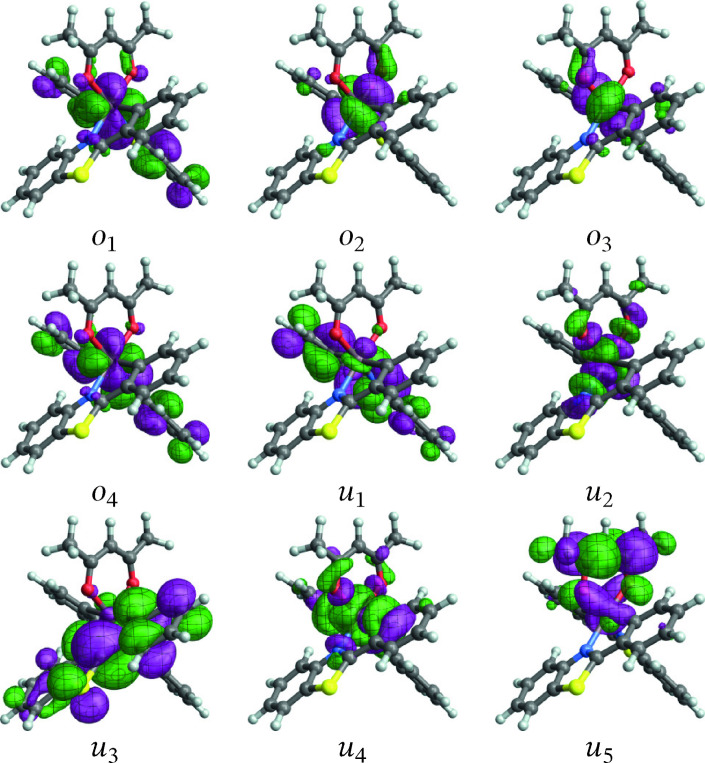
Orbitals comprising the (8,9) active space of [Ir(pbt)2(acac)] (**1**) from the state‐averaged triplet spin multiplicity calculation (the spin singlet orbitals appear very similar). Labels *o*/*u* are used to distinguish MOs that are occupied/unoccupied in the leading singlet ground‐state configuration.

**Figure 2:**
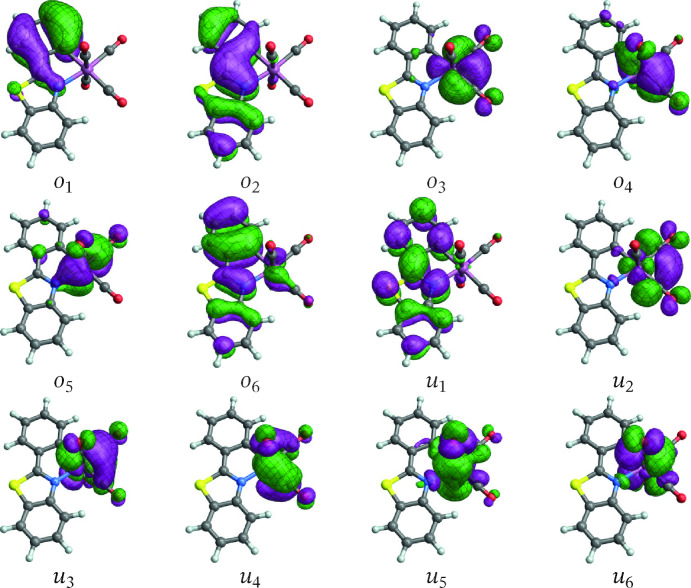
Orbitals comprising the (12,12) active space of [Re(CO)4(pbt)] (**2**) from the state‐averaged triplet spin multiplicity calculation (the spin singlet orbitals appear very similar). Labels *o*/*u* are used to distinguish MOs that are occupied/unoccupied in the leading singlet ground‐state configuration.

**Figure 3:**
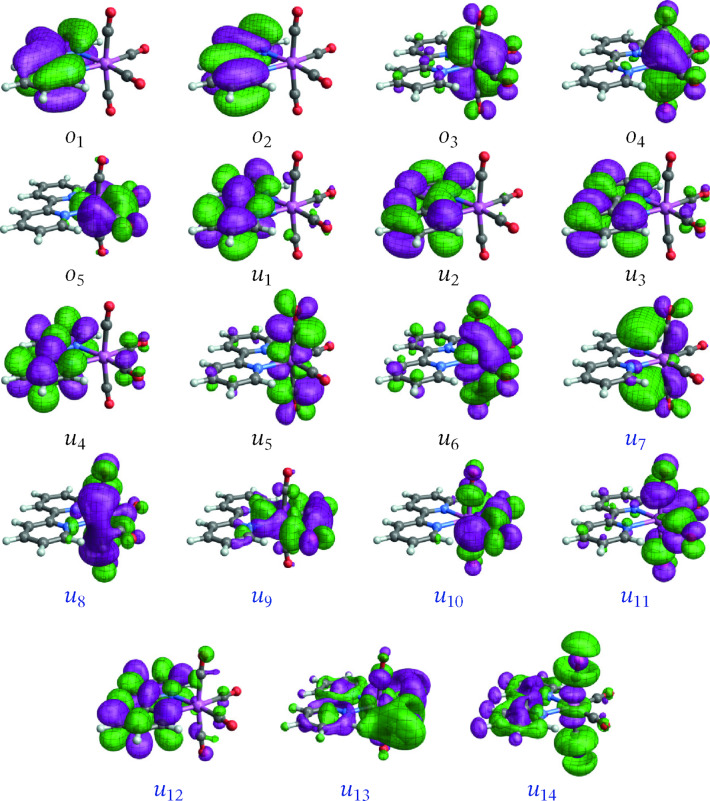
Orbitals comprising the (10,19) active space of [W(CO)4(bpy)] (**3**) from the state‐averaged singlet spin multiplicity calculation (the triplet states orbitals appear very similar). Labels *o*/*u* are used to distinguish MOs that are occupied/unoccupied in the leading singlet ground‐state configuration. Black labels are used for RAS2 orbitals and blue for RAS3.

**Figure 4:**
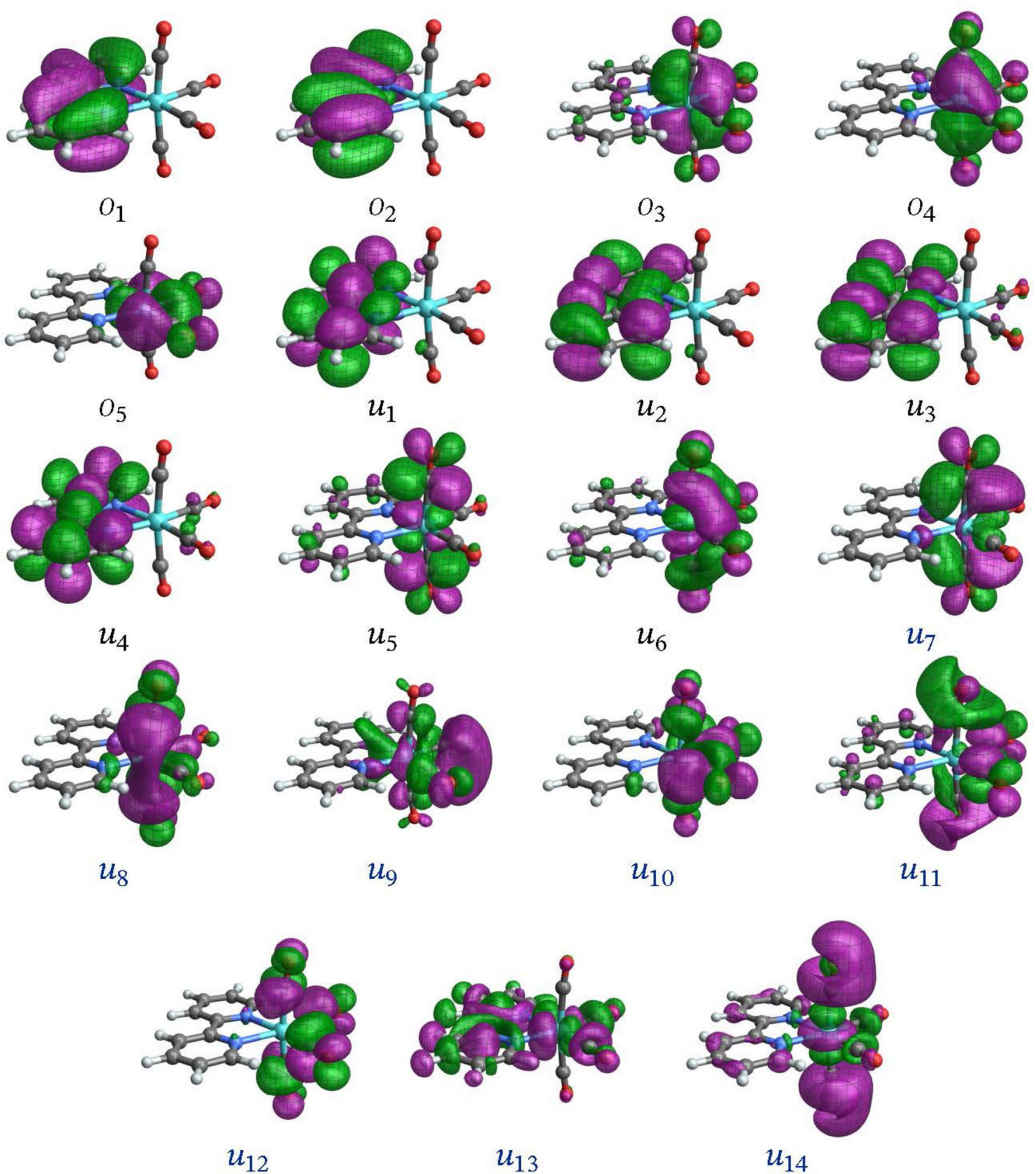
Orbitals comprising the (10,19) active space of [Mo(CO)4(bpy)] (**4**) from the state‐averaged singlet spin multiplicity calculation (the triplet states orbitals appear very similar). Labels *o*/*u* are used to distinguish MOs that are occupied/unoccupied in the leading singlet ground‐state configuration. Black labels are used for RAS2 orbitals and blue for RAS3.

Validation of the active spaces and wavefunction approaches entailed extensive test calculations and included comparisons of absorption spectra and state assignments with TD‐DFT results (present, and from the literature).^[6]^ Previous calculations for [Ir(ppy)_3_] indicated that the CASSCF level is sufficient for treating the low‐energy electronic states of this system.^[13]^ Likewise, CASSCF calculations were found to perform well for **1** and **2** and therefore we only report these results in the present work. Only the lowest triplet‐singlet transition was considered in detail for the two complexes. The orbitals with largest metal 5d contributions and the energetically closest ligand frontier orbitals were included in the active spaces (Figures 1 and 2), resulting in an 8 electrons in 9 orbitals (8,9) active space for **1** and a (12,12) active space for **2**. The active spaces of **3** (Figure 3) and **4** (Figure 4) have a more complicated setup, based on extensive test calculations. (Active space orbitals for the singlet state calculations are presented for **3** and **4**. The orbital sets for the triplet calculations are qualitatively the same.) Most of the relevant metal orbitals and the energetically lowest 2,2’‐bipyrimidine orbitals were included in the RAS2 space (unrestricted occupations). Orbitals with higher energy, unoccupied in the ground state, with large carbonyl ligand contributions were included in RAS3 allowing at most one electron.

The Amsterdam Density Functional^[41]^ (ADF) version 2021.102 package was employed for TD‐DFT calculations of the absorption spectra of **3** and **4**, using the all‐electron scalar zeroth‐order regular approximation (ZORA)^[42]^ relativistic Hamiltonian with the PBE functional and its hybrid variant (25 % exact exchange) PBE0,^[43]^ B3LYP, and the range‐separated exchange functional CAM−B3LYP.^[44]^ Slater‐type triple‐*ζ* polarized (TZP) basis sets were used for Mo and W, double‐*ζ* polarized (DZP) for C, O and N, and double‐*ζ* (DZ) for hydrogen.^[45]^ Calculations were performed with and without invoking the TDA.

For visualizations of orbitals, ‘cube’ format^[46]^ volume data files were generated from the program outputs with Pegamoid 2.7.^[47]^ Wolfram Mathematica^[48]^ version 13.0 was used to generate the isosurface graphics, using a notebook developed by one of us.^[49]^ Isosurfaces were generated at ±0.025 atomic units, which is not noted further. The imaginary parts of the SO‐NTOs were minimized according to the phase factor prescription put forward in Reference [13]. Residual imaginary parts were small and are therefore not discussed in detail in this work. The SF NTOs exhibited a predominant contribution from a single orbital pair in the respective transitions. Consequently, only these specific orbitals were selected for visualization. Transition dipole moments are given in atomic units (au), with 1 au corresponding to approximately 2.54 Debye (1 Debye=10^–18^ esu cm). A data repository for this study, containing Molcas inputs and outputs, and volume data for NTOs in ‘cube’ format, is available.^[50]^

## Results and Discussion

### Overview of Photophysical Data

The present study is focused on the spin‐forbidden emission from triplet spin states as the systems transition to the spin‐singlet ground state S_0_. A summary of the photophysical data is provided in Table 2. For each relevant transition, the vertical transition energy, the Einstein emission coefficient (*A*), and the electric dipole oscillator strength (*f*) are given, along with a brief characterization of the transition and the main theoretical method used to obtain the data. Furthermore, the zero‐field splitting (ZFS) for the triplet states is reported in Table 2 based on calculations using RASSCF and MC‐pDFT methods. Where available, experimental data are provided for comparison.

**Table 2:** Triplet‐singlet emission transitions considered in this study.

System	SF/SO^[a]^	Method^[b]^		Δ*E*/eV	ZFS/cm^−1^		*f*	*A*/s^–1^
			Calc.	Expt.	Calc.	Expt.		
**1**	$T_{1}\to S_{0}$	CASSCF	2.44	2.23^[c]^	145	103^*f*^	1.71E ‐3	4.44E5
	2→1	MC‐pDFT	1.67		677		2.04E ‐2	2.46E6
**2**	$T_{1}\to S_{0}$	CASSCF	2.93	2.50^[d]^	0.46	<2^[f]^	7.28E ‐5	2.70E4
	$3\left(4\right)\to 1$	MC‐pDFT	2.43		3.04		6.42E ‐5	1.65E4
**3**	$T_{1}\to S_{0}$	RASSCF	1.51	1.61^[e]^	62	–	1.87E ‐4	1.86E4
	3→1	MC‐pDFT	1.82		81		7.43E ‐3	1.07E6
	$T_{3}\to S_{0}$	RASSCF	1.74		71	–	2.01E ‐3	2.66E5
	$9\left(7\right)\to 1$	MC‐pDFT	1.79		1375		2.13E ‐4	2.96E4
**4**	$T_{1}\to S_{0}$	RASSCF	1.96	1.60^[e]^	9	–	1.07E ‐5	1.79E3
	$3\left(4\right)\left[4\right]\to 1$	MC‐pDFT	2.48		84		1.00E ‐3	2.66E5
		PT2	1.78		8		3.67E ‐4	5.04E4
	$T_{3}\to S_{0}$	RASSCF	2.05		22	–	2.14E ‐4	3.89E4
	$7\left(5\right)\left[8\right]\to 1$	MC‐pDFT	2.31		281		1.21E ‐5	2.81E3
		PT2	1.89		4		4.60E ‐5	7.14E3

[a] For a given transition, the first line (SF) is the assignment based on spin‐free states. The second line (SO) is the assignment in terms of the SO states. SO state number (1 is the ground state) in RASSCF calculation is given without brackets or parentheses. SO state numbering includes the individual triplet components; the provided emission intensities are based on the most intense triplet component for a given triplet state. SO state number in MC‐pDFT in parentheses. SO state number from PT2 is given in square brackets. For **3** and **4**, emission data are provided both for T_1_ and T_3_. [b] For a given transition, the first line gives the calculated and experimental vertical transition energy, ZFS, *f*, and *A* for the RASSCF SO calculation. Additional lines give data based on MC‐pDFT or PT2 SO calculations based on RASSCF reference states. [c] Reference [51]. [d] 0–0 transition. Reference [52]. [e] Reference [53]. [f] Reference [2].

The ordering of SO states and the corresponding transitions exhibit variations depending on the level at which the dynamic correlation is treated (or not). Accordingly, the SO state numbers for which the data are provided may vary as indicated. For the data listed in Table 2, the strongest‐emitting triplet state components were chosen for tabulating the *f* and *A* values. The calculated vertical transition energies are compared to available experimental data extracted from the emission spectra. For complexes **3** and **4**, two sets of emission data are provided because these systems have previously been considered to be cases that violate the Kasha rule.^[6]^

The RAS/CASSCF transition energies overestimate the experimental emission energy for **1**, **2**, and **4** by several tenths of an eV, whereas for **3** the transition energy is slightly underestimated. The deviations between the calculated and experimental energies are not necessarily a major concern because the calculated data are not corrected for vibronic effects, solvent effects, or the dynamics of the systems. Nonetheless, improved energies should be expected from calculations that take into account the dynamic electron correlation.

Complex **4** has the smallest electron count among the systems and is therefore amenable to RASPT2 benchmark calculations. As seen from Table 2, both the (close‐lying) transition energies from states T_1_ and T_3_ move closer to the experimental 1.60 eV reference value when the state energies are corrected with PT2. The residual deviations between calculated and experimental transition energies can easily be rationalized by the lack of vibronic and dynamic effects in the calculations in addition to the approximations made in the electronic structure model.

The other systems are rather large for correlated electronic structure calculations with the chosen active spaces. We tested MC‐pDFT in calculations of all complexes (see Table 2). With the exception of complex **2**, the MC‐pDFT transition energies are further away from the experimental reference values than the RASSCF energies. Furthermore, for several of the triplet states the ZFS from MC‐pDFT appears to be spuriously large. The slight overestimation of the ZFS with MC‐pDFT for complex **2** does not raise immediate concerns. The RASSCF and MC‐pDFT T_1_ transition energy for **3** bracket the experimental value, and although the MC‐pDFT energy appears potentially a bit too high, the ZFS is very similar to the RASSCF result. Therefore, RASSCF and MC‐pDFT perform similarly for treating the triplet emission of this system.

Comparing the values of oscillator strengths and emission coefficients for **3** and **4** indicates that the transition probabilities for the lowest triplet state increase at the MC‐pDFT and PT2 levels of theory, relative to the transition from state T_3_. Since there were previous assignments of these systems as anti‐Kasha emitters based on TD‐DFT/B3LYP, we provide a comparative state labeling in comparison to B3LYP states in the SI, Figures S9–S10. When accounting for dynamic correlation, the energy difference between T_1_ and S_1_ is usually diminished, resulting in a significant mixing of these spin states under the SO interaction for complex **1**. While changes in state energies due to dynamic correlation do not yield qualitative differences in the compositions of the SO states for the other complexes, they do impact the order of states, as mentioned (refer to Table 2). The compositions of the relevant spin‐orbit states, are detailed in Table 3 for RASSCF and MC‐pDFT calculations and in Table 4 for PT2.

**Table 3:** Composition of the spin‐orbit states.

		RASSCF				MC‐pDFT		
System	SO	*E*_SO_/eV	SF	*E*_SF_/eV	SO	*E*_SO_/eV	SF	*E*_SF_/eV
**1**	1	0.00	94.67 % S_0_	0.00	1	0.00	94.45 % S_0_	0.00
			2.28 % T_2_	3.37			2.50 % T_2_	3.21
			0.97 % T_6_	3.90			0.93 % T_6_	3.95
			0.51 % T_8_	4.03			0.49 % T_8_	4.09
	2	2.44	92.35 % T_1_	2.35	2	1.67	52.23 % T_1_	1.61
			2.61 % T_3_	3.47			42.72 % S_1_	1.65
			1.01 % T_17_	5.81			1.45 % T_3_	3.41
			0.75 % S_5_	4.11			0.59 % T_19_	5.64
**2**	1	0.00	99.98 % S_0_	0.00	1	0.00	99.97 % S_0_	0.00
			0.01 % T_5_	4.56			0.01 % T_5_	4.27
	3	2.93	99.91 % T_1_	2.93	4	2.43	99.93 % T_1_	2.43
			0.06 % T_7_	4.67			0.03 % T_7_	4.76
			0.03 % S_7_	4.98			0.03 % S_7_	4.49
**3**	1	0.00	99.94 % S_0_	0.00	1	0.00	99.95 % S_0_	0.00
			0.03 % T_1_	1.54			0.02 % T_1_	1.87
			0.03 % T_2_	1.90			0.02 % T_2_	2.02
	3	1.51	93.18 % T_1_	1.54	3	1.82	79.72 % T_1_	1.87
			4.06 % S_2_	1.97			17.74 % T_2_	2.02
			2.76 % T_3_	1.97			2.50 % S_3_	2.32
	9	1.74	82.72 % T_3_	1.71	7	1.79	55.60 % T_3_	1.71
			16.49 % T_1_	1.54			44.30 % T_1_	1.69
			0.78 % S_3_	2.17			0.10 % S_3_	2.20
**4**	1	0.00	100.0 % S_0_	0.00	1	0.00	100.0 % S_0_	0.00
	3	1.96	99.54 % T_1_	1.96	4	2.48	96.13 % T_1_	2.48
			0.28 % S_2_	2.46			3.45 % T_2_	2.62
			0.18 % T_3_	2.46			0.42 % S_3_	2.83
	7	2.05	98.03 % T_3_	2.05	5	2.31	71.57 % T_3_	2.33
			1.88 % T_1_	1.86			27.43 % S_1_	2.36
			0.09 % S_3_	2.65			0.99 % T_2_	2.60

**Table 4:** Composition of the RASSCF spin‐orbit states for complex [Mo(CO)_4_(bpy)] in comparison to RASPT2.

			RASSCF				PT2	
System	SO	*E*_SO_/eV	SF	*E*_SF_/eV	SO	*E*SO/eV	SF	*E*_SF_/eV
4	1	0.00	100.0 % S0	0.00	1	0.00	100.0 % S0	0.00
	3	1.96	99.54 % T1	1.96	4	2.48	97.10 % T1	1.78
			0.28 % S2	2.46			2.37 % T3	1.93
			0.18 % T3	2.46			0.17 % S3	2.32
	7	2.05	98.03 % T3	2.05	8	1.89	68.12 % T3	1.87
			1.88 % T1	1.86			31.86 % T1	1.85
			0.09 % S3	2.65			0.03 % S3	2.36

We additionally conducted an extensive assessment of large restricted active spaces for higher triplets in **3** and **4**. This involved calculating absorption spectra using both TD‐DFT and RASSCF/MC‐pDFT/PT2 approaches (see SI Figures S6, S7 and S8) and comparisons with experimental data from Reference [54]. Among the TD‐DFT calculations, CAM−B3LYP exhibited the closest agreement with experimental absorption spectra, serving as a comparison for the RASSCF, MC‐pDFT, and PT2 calculations. Upon analysis of excitations and orbitals, it became evident that the energetic order of states in RASSCF and TD‐DFT/CAM−B3LYP calculations demonstrated good correspondence, unlike TD‐DFT calculations with the B3LYP functional which also underestimated the excited states energies. Additionally, the incorporation of dynamic correlation in RASSCF led to an improvement correspondence between the calculated absorption spectrum vs. experimental data and CAM−B3LYP. Based on the present calculations at the CAS/RAS and TD‐DFT levels, the emitting state for complexes **3** and **4** can be identified as T_1_.

To summarize thus far, similar to the previously^[13]^ investigated complex [Ir(ppy)_3_], a treatment of the emission at the CAS/RASSCF levels appears to be appropriate for complexes **1** and **3**. Complex **2** benefits from an improved emission energy when calculated with MC‐pDFT, and **4** is reasonably well described at the RASSCF and RASPT2 levels (the energetic corrections for **4** are not dramatic and RASSCF can be considered as a reasonable approach for studying the emission in this system). We therefore proceed with an analysis of the NTOs and SO‐NTOs based on CASSCF calculation for complex **1**, RASSCF for **3**, XMS‐RASPT2 (‘PT2’) for **4**, and CASSCF/MC‐pDFT calculations for **2**. Additional emission data for the complexes calculated at other levels of theory can be found in the SI.

### Natural Transition Orbitals

**Figure 5:**
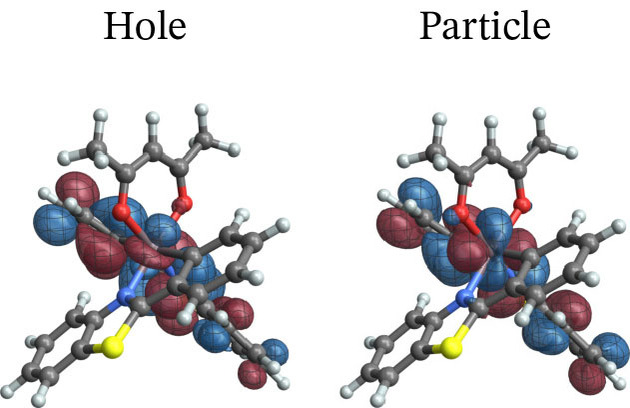
Spin‐free NTOs for the $T_{1}\to S_{0}$ transition of [Ir(pbt)2(acac)].

**[Ir(pbt)2(acac)]**: Spin‐free (SF) NTOs for the $T_{1}\to S_{0}$ spin‐forbidden transition of [Ir(pbt)2(acac)] (**1**) are shown in Figure 5. A comparison with the active‐space orbitals of the system in Figure 1 reveals that the ‘particle’ NTO corresponds to the HOMO (*o*_4_ in Figure 1) and the ‘hole’ corresponds to the LUMO (*u*_1_). In keeping with the convention adopted in Reference [13], for emission transitions the the hole NTO is occupied/empty in the excited/ground state, and the particle NTO is occupied/empty in the ground/excited state. The SF NTOs yield a straightforward assignment of the transition as predominantly ligand‐centered π*–*π* (LUMO to HOMO) emission with involvement of metal 5d AOs. The telltale d‐orbital shapes are clearly visible in Figure 1 as well as in Figure 5. As a reminder, the complex geometry was optimized for the T_1_ state. It appears that the emission localizes on one of the pbt ligands.

**Figure 6:**
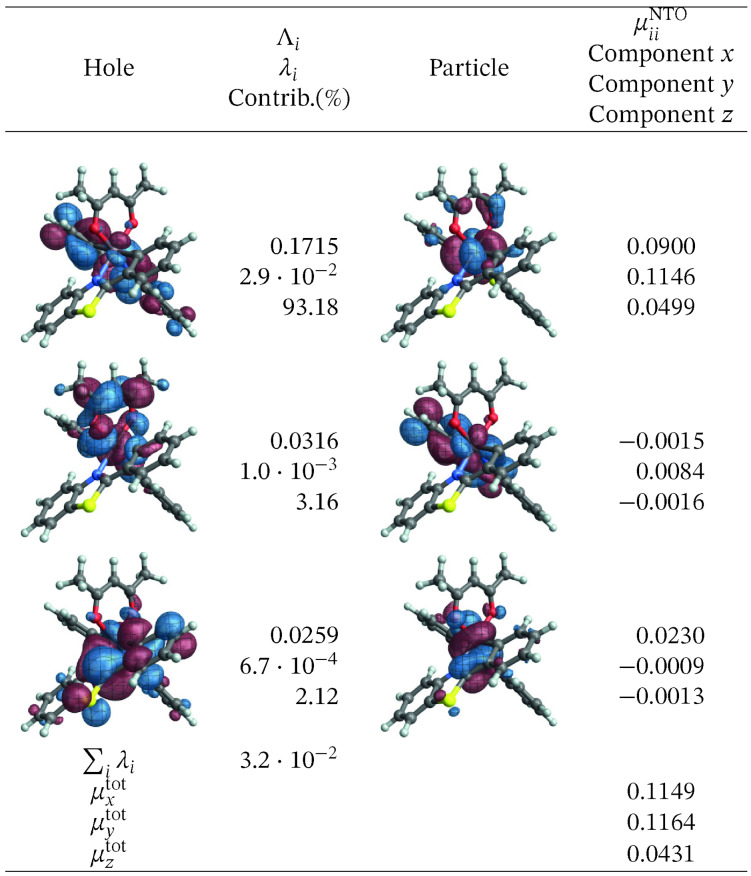
SO‐NTOs for the 2→1 transition of [Ir(pbt)2(acac)] (CASSCF calculation).

The corresponding SO‐NTOs are shown in Figure 6. The SO‐NTOs were generated for the transition between SO states 2 and 1, which is the component of the T_1_ emission that carries the highest intensity in the calculation. Per Table 3, SO state 2 derives from the T_1_ triplet with notable admixtures of other triplets and the S_5_ singlet state. State 1 corresponds to S_0_ with admixtures of T_2_ and higher triplets. In Figure 6, the three largest contributions to the transition density matrix are shown, along with the SO‐NTO pair contributions to the transition dipole moment (TDM) and the total calculated TDM. The Cartesian components of the TDM are given in reference to the coordinate system used to represent the optimized geometry of the complex (see Section S1 in the SI where the XYZ coordinates used for the calculations are listed).

**Figure 7:**
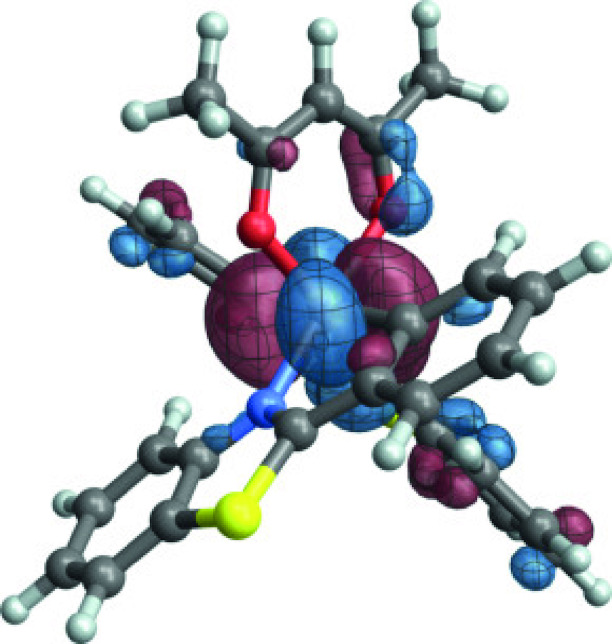
A linear combination of active space orbitals *o*_1_, *o*_2_ and *o*_3_ of [Ir(pbt)2(acac)] (Figure 1) resembling the particle SO‐NTO for the emission of **1** (Figure 6) associated with most of the dipole intensity.

We reiterate that the SO‐NTOs are constructed from the same transition density matrix that generates the dipole oscillator strength of the spin‐forbidden transition. Therefore, it is not surprising that the SO‐NTO pairs are different from the SF NTOs of Figure 5. For the largest contribution, creating most of the transition dipole moment, the hole NTO is seen to be very similar to the SF hole NTO, corresponding to the LUMO. However, the accompanying particle SO‐NTO corresponds to a non‐bonding Ir 5d orbital that is occupied in the ground state. The intensity is therefore generated by the SO‐induced admixture of a spin‐allowed ligand‐to‐metal charge transfer (LMCT) into the spin‐forbidden transition. The orbitals can be associated with the $S_{5}\to S_{0}$ SF transition, which involves the orbitals *u*_1_ and *o*_1_, *o*_2_, *o*_3_ of the active space. Figure 7 shows that a linear combination of the three relevant occupied orbitals (a 50–50 % normalized linear combination of *o*_1_ and *o*_3_, then taking a 50–50 % normalized linear combination of the result with *o*_2_) closely resembles the SO‐NTO, and likewise a linear combination of the SF particle NTOs produces a plot with the same appearance (Figure S15).

In the second largest SO‐NTO pair contribution to the TDM of the emission, the particle closely corresponds to the HOMO whereas the hole in the excited state has metal 5d and acac π* contributions. This picture is indicative of the SO‐induced spin‐allowed de‐excitation from antibonding metal‐centered 5d and acac π* orbitals. The third listed SO‐NTO pair involves emission from the second pbt ligand into a mainly metal‐centered orbital. The contributions of the second and the third‐largest SO‐NTO pair to the TDM are small, however. Any modifications of the complex with the aim to increase the TDM of the T_1_ emission further – for example, by ligand substitution or functionalization – would likely have to target the spin‐allowed LMCT component in the transition corresponding to the first SO‐NTO pair in Figure 6.

We note in passing that the treatment of dynamic correlation with MC‐pDFT leads to a very large (43 %) admixture of the S_1_ state in SO state 2. Accordingly, we see more resemblance of the corresponding SO‐NTOs (Figure S14) with the SF NTOs (Figure 5) than it is the case for the CASSCF results in Figure 6. As discussed already, however, the MC‐pDFT calculation for this transition appears to be deficient.

**Figure 8:**
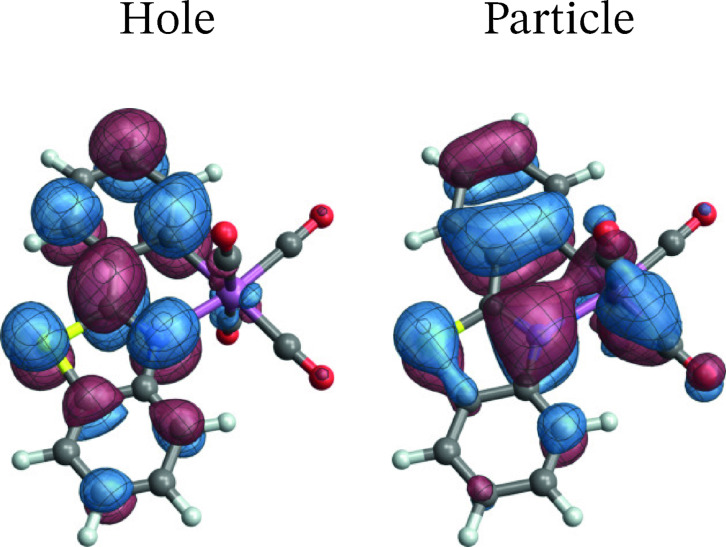
Spin‐free NTOs for the $T_{1}\to S_{0}$ transition of [Re(CO)4(pbt)]..

**Figure 9:**
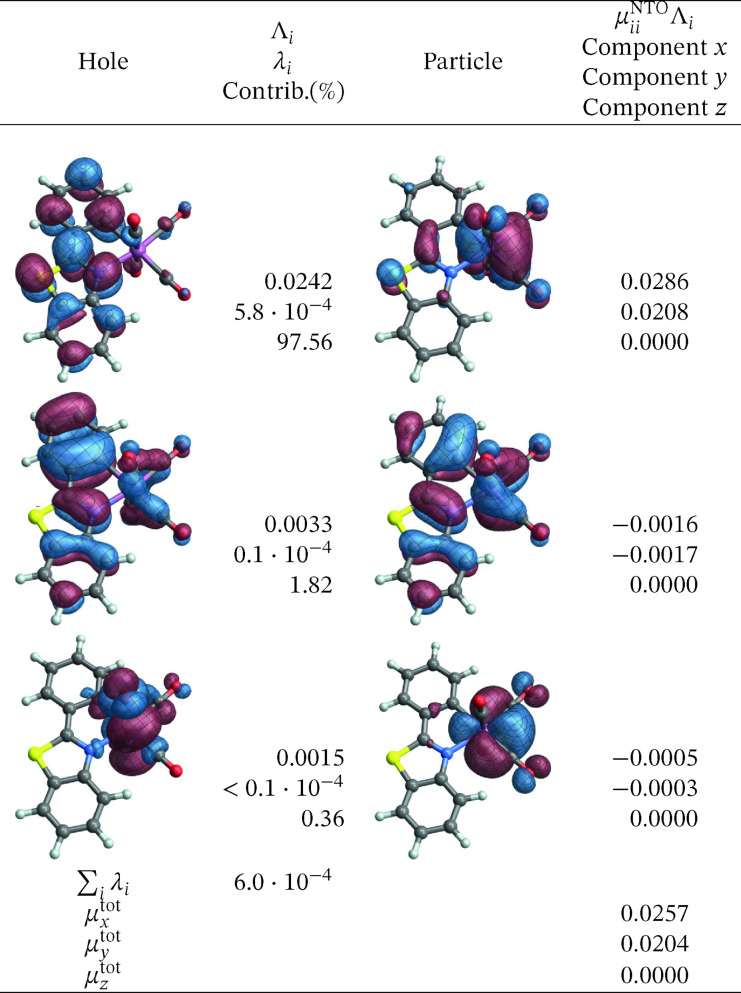
SO‐NTOs for the 4→1 transition of [Re(CO)4(pbt)] (CASSCF/MC‐pDFT calculation).

**[Re(CO)4(pbt)]**: Figure 8 shows the SF NTOs for the T_1_ emission of [Re(CO)4(pbt)]. The SF NTOs give the major assignment of the spin‐forbidden transition as LUMO to HOMO, which is evident from the comparison of the NTOs with the active space orbitals in Figure 2 (*u*_1_, *o*_6_). These orbitals are mainly localized in the pbt ligand but evidence, especially in the case of the HOMO‐like particle NTO, involvement of the Re 5d shell. As it was discussed already, the emission of this system appears to be better described at the MC‐pDFT than the RAS/CASSCF level. The corresponding SO‐NTOs for the transition between SO states 4 and 1 (cf. Table 3), which is the most intense triplet component at this level of calculation, are displayed in Figure 9.

As in the case of complex **1**, the intensity of the emission is not associated with the same pair of NTOs that assigns the transition in the SF case. Instead, the intensity is carried by a hole SO‐NTO that essentially represents the LUMO, and a particle SO‐NTO composed of a Re 5d AO with bonding admixtures of carbonyl π*. The particle SO‐NTO can be thought of as a linear combination of the active space orbitals *o*_4_ and *o*_5_ in Figure 2. It is occupied in the ground state and represents one of the carbonyl back‐donation channels in the complex (another one is seen in the particle SO‐NTO of the second largest contribution). In other words, the intensity of the spin‐forbidden transition is generated by SO‐induced admixture of spin‐allowed LMCT (pbt‐centered π* to Re 5d) into the de‐excitation transition from T_1_. Also, as in the case of **1**, the SO‐NTOs for the analyzed transition of **2** gives almost all of the TDM from one SO‐NTO pair, with only minor additional contributions from other pairs of SO‐NTOs. The intensity‐generating orbitals can be associated mostly with $S_{7}\to S_{0}$ spin free transition involving *u*_1_, *o*_4_ and *o*_5_ orbitals of the active space.

**Figure 10:**
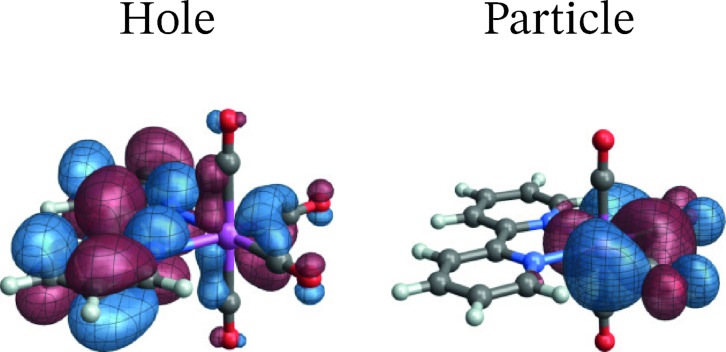
Spin‐free NTOs for the $T_{1}\to S_{0}$ transition of [W(CO)4(bpy)].

**Figure 11:**
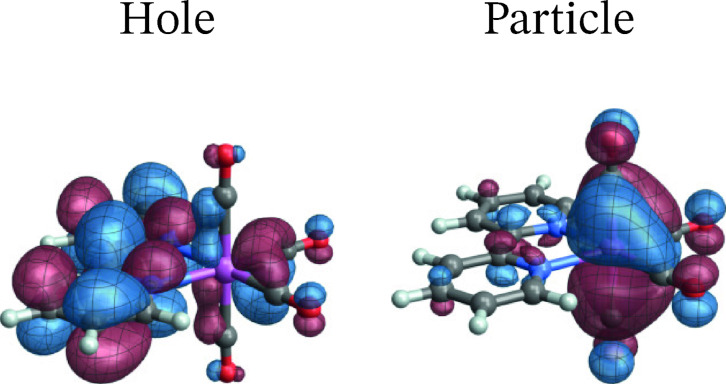
Spin‐free NTOs for the $T_{3}\to S_{0}$ transition of [W(CO)4(bpy)].

**[W(CO)4(bpy)]**: The spin‐free NTOs of complex **3** for the $T_{1}\to S_{0}$ and $T_{3}\to S_{0}$ spin‐forbidden transitions are shown in Figures 10 and 11, respectively. The SF NTOs identify closely with corresponding orbitals in the active space (Figure 3). Specifically, the LUMO (*u*_1_), a π* orbital of the bpy ligand delocalized toward the equatorial CO ligands is the hole for both de‐excitation transitions. The particle NTO for the T_1_ emission corresponds to the HOMO (*o*_5_ in Figure 3), which is a W 5d AO back‐donating into the in‐plane π* orbitals of the equatorial CO ligands. For the T_3_ emission, the particle NTO is a W 5d AO that is back‐donating into all four carbonyl ligands, corresponding to the *o*_4_ active space orbital in Figure 3. In other words, both transitions are assigned as LMCT with some involvement of the carbonyl ligands.

**Figure 12:**
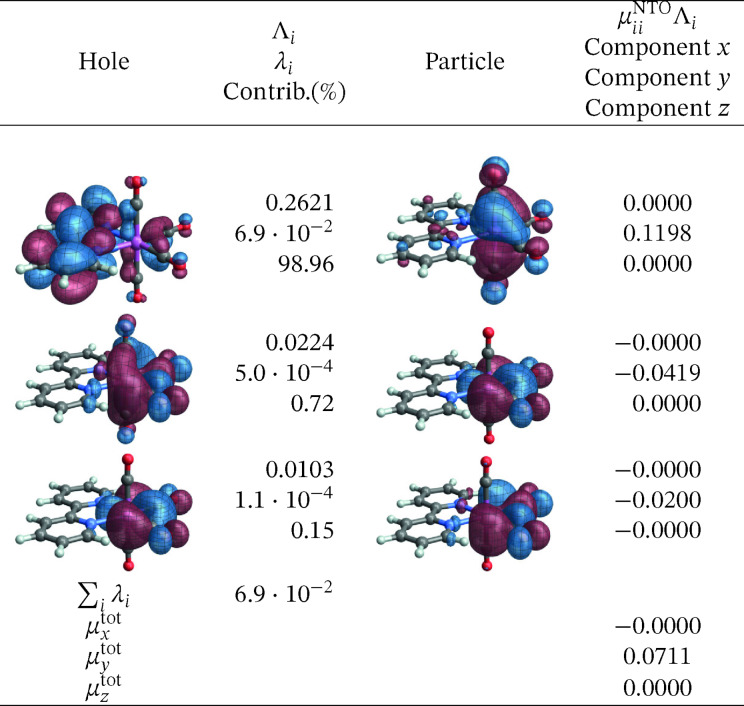
SO‐NTOs for the 3→1 transition of [W(CO)4(bpy)] (RASSCF calculation).

**Figure 13:**
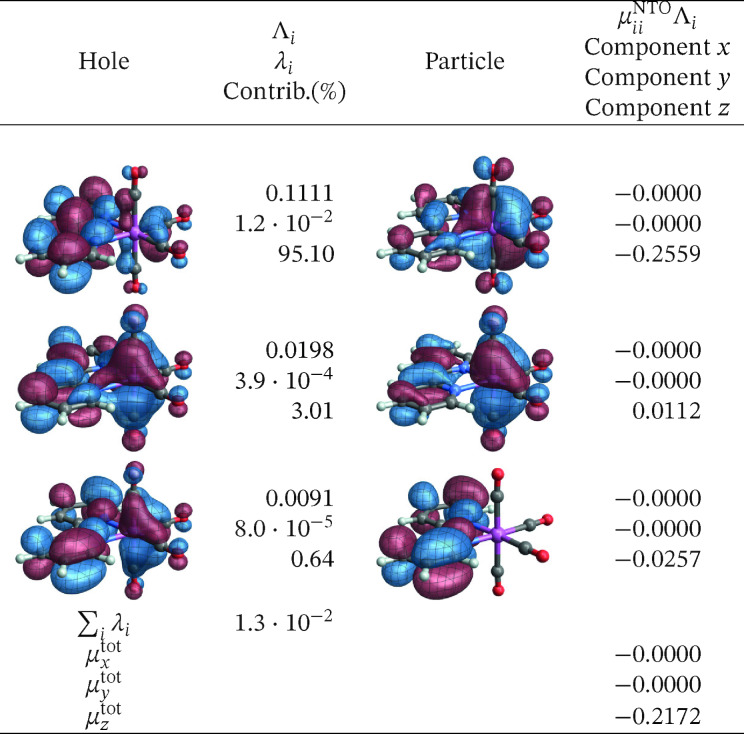
SO‐NTOs for the 9→1 transition of [W(CO)4(bpy)] (RASSCF calculation).

As mentioned, MC‐pDFT produced a spuriously large ZFS for the T_1_ state of this system, and we proceed with an analysis of the RASSCF calculation. The SO‐NTOs for the most intense components of the T_1_ and T_3_ emission at the SO level are shown in Figure 12 and 13, respectively. Interestingly, the SO‐NTO pair that generates the TDM of the spin‐forbidden T_1_ emission is qualitatively the same as the pair of SF NTOs for the de‐excitation from T_3_. This is indicative of a spin‐allowed LMCT involving another W 5d AO with carbonyl back‐donation mixing into the emission from T_1_ to generate the intensity. The particle SO‐NTO that generates most of the TDM in the T_3_ emission coincides neither with the T_1_ nor the T_3_ emission SF NTOs but represents a third CO back‐donating 5d AO (a close match with *o*_3_ in Figure 3). A secondary contribution (3 %) involves one of the other 5d orbitals as the particle but has a different hole SO‐NTO.

The SO‐NTO analysis therefore paints the following picture: The ground state features three occupied W 5d AOs with considerable back‐donation into carbonyl π* orbitals. In an octahedral complex, these 5d AOs would form the t_2g_ set. Each emission from one of the low‐energy triplet excited states can be assigned as LMCT from a bpy π* into one of the backbonding 5d orbitals of t_2g_ parentage. The intensities of these transitions are caused mainly by SO‐induced admixtures of spin‐allowed LMCT involving the same bpy π* but another one of the t_2g_ parentage orbitals. From the present as well as previous calculations on the system it is difficult to determine the energetic ordering of the bpy π* to 5d LMCT transitions.

**Figure 14:**
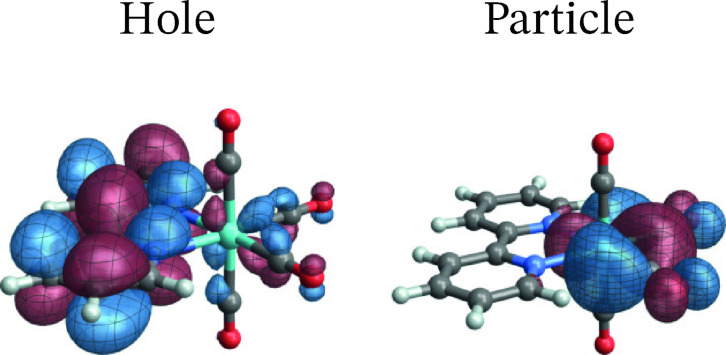
Spin‐free NTOs for the $T_{1}\to S_{0}$ transition of [Mo(CO)4(bpy)].

**Figure 15:**
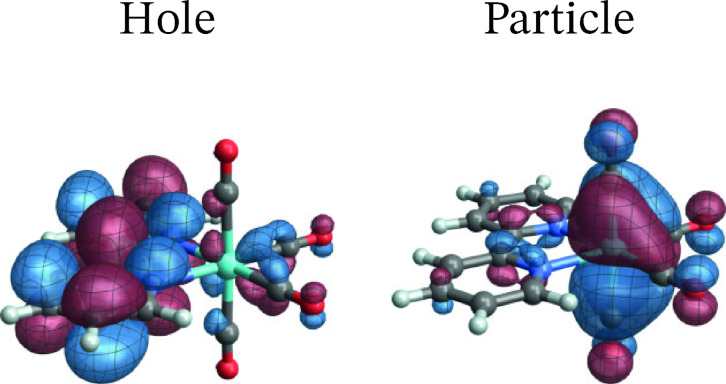
Spin‐free NTOs for the $T_{3}\to S_{0}$ transition of [Mo(CO)4(bpy)].

**[Mo(CO)4(bpy)]**: The transition analysis of **4** follows that of the tungsten analog **3** closely. The SF NTOs for the emission from T_1_ and T_3_ are shown in Figures 14 and 15, respectively. As it is the case for the tungsten complex, the SF NTOs are equivalent to the corresponding orbitals in the active space (Figure 4), namely, the LUMO (*u*_1_) representing the hole and one of the metal‐centered back‐bonding 4d AOs of t_2g_ parentage as the particle NTO. The SO‐NTO analysis in Figures S19 and S20 also reveals similar origins of the intensity as in the corresponding tungsten complex (but with 4d instead of 5d), such that we forego a detailed description. Note that the SO state assignment becomes notably different in the MC‐pDFT calculations, as discussed in Section S4 in the SI.

**Figure 16:**
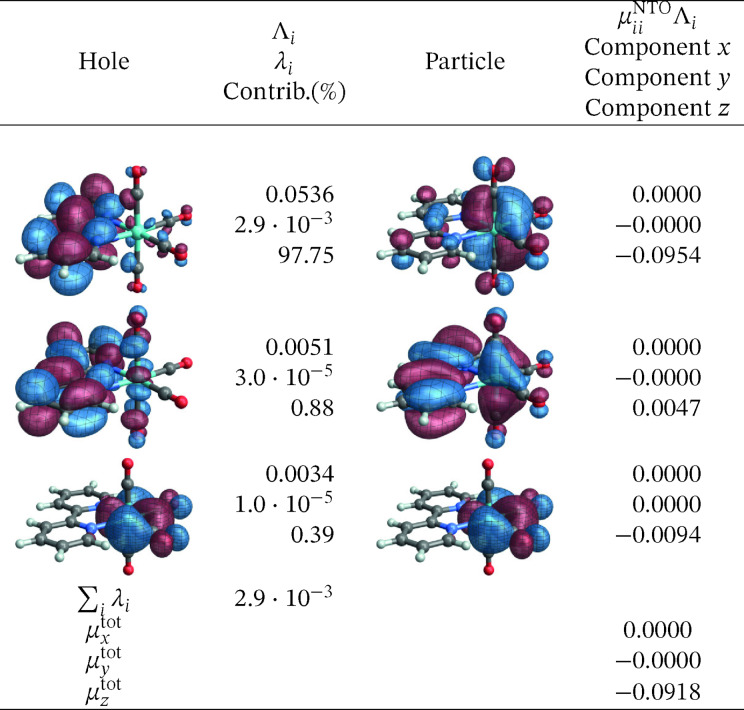
SO‐NTOs for the 4→1 transition of [Mo(CO)4(bpy)] (RASPT2 calculation).

**Figure 17:**
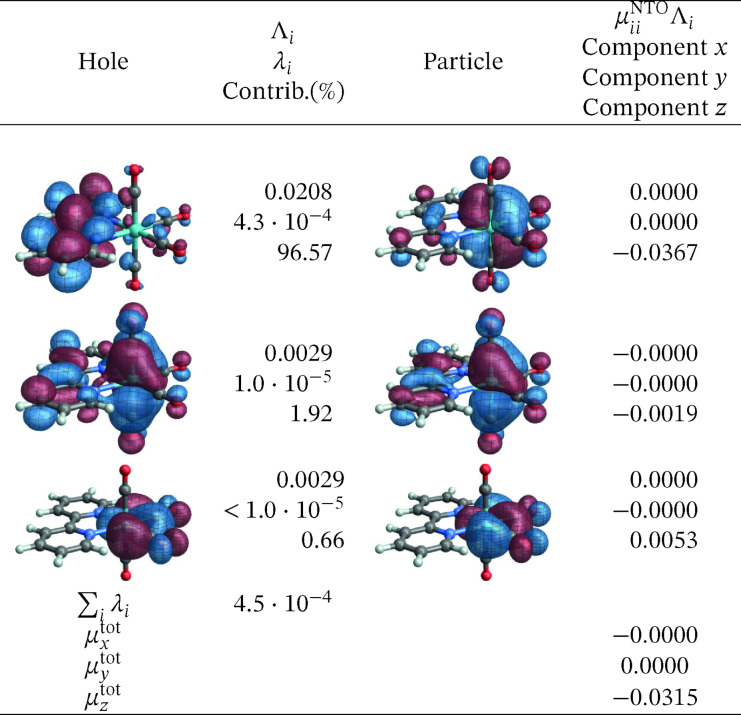
SO‐NTOs for the 8→1 transition of [Mo(CO)4(bpy)] (RASPT2 calculation).

As mentioned, the best results for the transition energies for this system were obtained from RASPT2 calculations. The corresponding SO‐NTOs are presented in Figures 16 and 17. The SO‐NTOs can be interpreted similarly as for the W complex: The ground state features three occupied M 4d AOs with considerable back‐donation into carbonyl π* orbitals. In an octahedral complex, these 4d AOs would form the t_2g_ set. Each emission from one of the low‐energy triplet excited states can be assigned as LMCT from a bpy π* into one of the backbonding 4d orbitals of t_2g_ parentage. The intensities of these transitions are caused mainly by SO‐induced admixtures of spin‐allowed LMCT involving the same bpy π* but another one of the t_2g_ parentage orbitals. The same SO‐NTO pair is the predominant source of intensity for both considered transitions at the SO level.

## Summary and Conclusions

Spin‐orbit natural transition orbital (SO‐NTO) methodology was recently developed in our group for complete and restricted active space (CAS/RAS) wavefunction calculations.^[13]^ In the present study, the technique was applied to analyze triplet‐to‐singlet emission in a set of transition metal complexes with Mo, W, Re, and Ir. The source of intensity of the spin‐forbidden electronic transitions that underlie the emission of the complexes was revealed in an intuitive manner by the SO−NTOs. In simple cases, the appearances of the SO‐NTOs can be rationalized via the SO‐induced admixtures of transitions among SF states of the same multiplicity into the T_1_‐S_0_ emission. In such cases, spin‐free NTOs can be used additionally to characterize the intensity. In more complicated cases it may be preferable to analyze the electronic intensity of a spin‐forbidden transition directly with SO‐NTOs. This approach also allows to analyze explicitly which of the triplet components is responsible for most of the intensity, or if they all contribute.

## Conflict of interests

The authors declare no conflict of interest.

1

## Supporting information

As a service to our authors and readers, this journal provides supporting information supplied by the authors. Such materials are peer reviewed and may be re‐organized for online delivery, but are not copy‐edited or typeset. Technical support issues arising from supporting information (other than missing files) should be addressed to the authors.

Supporting Information

## Data Availability

The data that support the findings of this study are available in the supplementary material of this article.
