# Supplementary material for: Triplet‐Singlet Emission of d‐Block Metal Complexes Characterized by Spin‐Orbit Natural Transition Orbitals
Source: ChemistryOpen. 2024 Mar 5;13(8):e202300291. doi: 10.1002/open.202300291 (PMC11319217; doi:10.1002/open.202300291)
Supplement: Supplementary file 1 — Supporting Information [file OPEN-13-e202300291-s001.pdf]

ChemistryOpen

Supporting Information

Triplet-Singlet Emission of d-Block Metal Complexes Characterized by Spin-Orbit Natural Transition Orbitals

A. Zaichenko and J. Autschbach*

S1 Molecular Structures

Table S1: UDFT structure for T_1 triplet state of $[Ir(pbt)_2(acac)]$.

Atom Nr.	*x*	*y*	*z*
77	-0.103168	0.632882	-0.062370
6	-2.356803	-1.113355	-0.466073
16	-3.213066	-2.367882	-1.368548
1	-2.358904	-4.394911	-3.468712
1	4.957211	0.116281	-0.751044
1	1.412040	-3.753843	5.134351
6	-2.795766	-0.391130	0.640247
6	-4.089381	-0.505766	1.247438
6	-4.389075	0.266825	2.354972
6	-3.433463	1.161124	2.890078
6	-2.161263	1.277336	2.295471
6	-1.802729	0.530090	1.176113
6	-1.320617	3.138119	-1.078942
6	1.024540	4.430434	1.708274
1	-5.373319	0.182577	2.824315
7	1.263463	-0.535686	0.953481
1	-1.436796	1.971767	2.733095
6	-0.591447	3.918080	-0.160487
6	0.326388	3.448816	0.797564
7	-1.014021	-0.923944	-0.928176
1	0.915564	2.039982	-2.976453
6	-2.286647	3.808324	-2.026270
8	-1.259024	1.871225	-1.208000
6	2.516337	-0.408774	0.513022
16	3.694462	-1.281872	1.458199
1	-2.250828	4.903327	-1.959485
1	-4.827561	-1.203853	0.842527
1	-0.967710	-1.362422	2.422983
6	2.788079	0.358367	-0.681223
6	4.071123	0.535597	-1.237795

6	4.200501	1.255020	-2.420930
6	3.054426	1.788979	-3.036062
6	1.787235	1.612655	-2.471185
6	1.609738	0.891641	-1.277262
1	-3.686477	1.762290	3.767720
8	0.648619	2.230297	0.991620
1	3.159732	2.349346	-3.970134
1	5.185672	1.403362	-2.869498
1	1.757315	-3.133442	-3.905487
1	-0.759531	4.993958	-0.192060
1	0.709901	5.466994	1.531592
1	2.113461	4.350823	1.560322
1	0.822609	4.161873	2.757514
1	-3.308739	3.465569	-1.797006
1	-2.067085	3.494331	-3.058635
6	1.164225	-1.370176	2.066237
6	2.413456	-1.875040	2.498938
6	2.522961	-2.728973	3.599791
6	1.354503	-3.085705	4.271891
6	0.106564	-2.597982	3.845866
6	-0.003262	-1.745307	2.750194
1	-0.798628	-2.892036	4.381944
1	3.495290	-3.108473	3.920769
6	-1.721233	-2.738798	-2.241065
6	-0.662291	-1.852380	-1.865014
6	0.602277	-2.015430	-2.480474
6	0.778488	-3.015375	-3.434548
6	-0.273693	-3.869310	-3.795620
6	-1.536230	-3.731680	-3.192266
1	-0.116437	-4.647221	-4.546249
1	1.420324	-1.354218	-2.205692

Table S2: TD-DFT structure for T_1 triplet state of $[Re(CO)_4(pbt)]$.

Atom Nr.	*x*	*y*	*z*
75	0.986980	0.421073	0.000000

6	-0.547404	-2.267949	0.000000
6	1.842113	-2.736805	0.000000
6	-0.823068	-3.685105	0.000000
6	-1.540066	-1.297756	0.000000
6	1.565087	-4.121256	0.000000
1	2.890504	-2.423952	0.000000
6	0.221047	-4.581495	0.000000
1	-1.857542	-4.038940	0.000000
16	-3.298162	-1.586341	0.000000
1	2.385399	-4.843111	0.000000
1	0.016703	-5.655412	0.000000
6	-2.301225	0.859692	0.000000
6	-3.556970	0.147414	0.000000
6	0.836589	-1.774856	0.000000
7	-1.203695	0.077569	0.000000
6	-2.334669	2.286523	0.000000
6	-4.772876	0.819226	0.000000
8	0.973736	0.227527	-3.157215
6	0.971472	0.314360	-2.015802
6	1.009515	2.404159	0.000000
6	0.971472	0.314360	2.015802
6	2.935630	0.415919	0.000000
8	1.067171	3.558616	0.000000
8	0.973736	0.227527	3.157215
8	4.088962	0.381567	0.000000
6	-3.554297	2.940815	0.000000
6	-4.770784	2.222780	0.000000
1	-5.720014	2.762968	0.000000
1	-3.574898	4.032861	0.000000
1	-1.404043	2.849138	0.000000
1	-5.714074	0.265775	0.000000

Table S3: TD-DFT (TDA) structure for T_1 triplet state of $[Re(CO)_4(pbt)]$.

Atom Nr.	*x*	*y*	*z*
75	0.984215	0.410838	0.000000

6	-0.549206	-2.269311	0.000000
6	1.846291	-2.736051	0.000000
6	-0.820712	-3.682859	0.000000
6	-1.540309	-1.294248	0.000000
6	1.571192	-4.113878	0.000000
1	2.893267	-2.419120	0.000000
6	0.225912	-4.575450	0.000000
1	-1.853679	-4.040559	0.000000
16	-3.293734	-1.580885	0.000000
1	2.390655	-4.836584	0.000000
1	0.024602	-5.649969	0.000000
6	-2.299464	0.862396	0.000000
6	-3.553634	0.153135	0.000000
6	0.835661	-1.773799	0.000000
7	-1.201438	0.074490	0.000000
6	-2.334814	2.286426	0.000000
6	-4.771605	0.822870	0.000000
8	0.954987	0.223559	-3.159312
6	0.960278	0.307619	-2.018198
6	1.014972	2.398250	0.000000
6	0.960278	0.307619	2.018198
6	2.934267	0.408777	0.000000
8	1.082056	3.551379	0.000000
8	0.954987	0.223559	3.159312
8	4.087771	0.378121	0.000000
6	-3.555463	2.940577	0.000000
6	-4.771654	2.224375	0.000000
1	-5.720941	2.764330	0.000000
1	-3.575280	4.032750	0.000000
1	-1.405698	2.851598	0.000000
1	-5.711428	0.266935	0.000000

Table S4: TD-DFT structure for T_1 triplet state of $[W(CO)_4(bpy)]$.

Atom Nr.	*x*	*y*	*z*
74	-0.000000	0.000000	-0.775001

6	0.000000	-0.718426	2.255759
6	0.000000	-2.693886	0.966762
6	0.000000	-1.513830	3.436927
6	-0.000000	0.718426	2.255759
6	0.000000	-3.513204	2.086325
1	0.000000	-3.134051	-0.033550
6	0.000000	-2.889614	3.356860
1	0.000000	-1.028748	4.412524
6	-0.000000	1.513830	3.436927
1	0.000000	-4.597166	1.973021
1	0.000000	-3.490748	4.269170
6	-0.000000	2.693886	0.966762
6	-0.000000	2.889614	3.356860
1	-0.000000	1.028748	4.412524
6	-0.000000	3.513204	2.086325
1	-0.000000	3.134051	-0.033550
1	-0.000000	3.490748	4.269170
1	-0.000000	4.597166	1.973021
6	-2.055311	-0.000000	-0.754371
6	-0.000000	1.417880	-2.236350
6	2.055311	0.000000	-0.754371
6	0.000000	-1.417880	-2.236350
8	-0.000000	2.231559	-3.055605
8	3.204922	0.000000	-0.784816
8	0.000000	-2.231559	-3.055605
8	-3.204922	-0.000000	-0.784816
7	-0.000000	1.352619	1.024223
7	0.000000	-1.352619	1.024223

Table S5: TD-DFT (TDA) structure for T_1 triplet state of $[W(CO)_4(bpy)]$.

Atom Nr.	*x*	*y*	*z*
74	-0.000000	0.000000	-0.775202
6	0.000000	-0.718537	2.255782
6	0.000000	-2.694039	0.967126
6	0.000000	-1.513709	3.437092

6	-0.000000	0.718537	2.255782
6	0.000000	-3.513405	2.086900
1	0.000000	-3.134235	-0.033183
6	0.000000	-2.889586	3.357138
1	0.000000	-1.028579	4.412652
6	-0.000000	1.513709	3.437092
1	0.000000	-4.597376	1.973747
1	0.000000	-3.490537	4.269579
6	-0.000000	2.694039	0.967126
6	-0.000000	2.889586	3.357138
1	-0.000000	1.028579	4.412652
6	-0.000000	3.513405	2.086900
1	-0.000000	3.134235	-0.033183
1	-0.000000	3.490537	4.269579
1	-0.000000	4.597376	1.973747
6	-2.055096	-0.000000	-0.754632
6	-0.000000	1.416903	-2.237272
6	2.055096	0.000000	-0.754632
6	0.000000	-1.416903	-2.237272
8	-0.000000	2.229985	-3.057124
8	3.204756	0.000000	-0.785396
8	0.000000	-2.229985	-3.057124
8	-3.204756	-0.000000	-0.785396
7	-0.000000	1.353019	1.024575
7	0.000000	-1.353019	1.024575

Table S6: TD-DFT structure for T_3 triplet state of [W(CO)$_4$(bpy)].

Atom Nr.	*x*	*y*	*z*
74	-0.000000	0.000000	-0.761577
6	0.000000	-0.718218	2.264315
6	0.000000	-2.698971	0.977206
6	0.000000	-1.510911	3.447660
6	-0.000000	0.718218	2.264315
6	0.000000	-3.513688	2.098737
1	0.000000	-3.134748	-0.024769

6	0.000000	-2.886696	3.369140
1	0.000000	-1.025163	4.422958
6	-0.000000	1.510911	3.447660
1	0.000000	-4.598200	1.989817
1	0.000000	-3.486415	4.282485
6	-0.000000	2.698971	0.977206
6	-0.000000	2.886696	3.369140
1	-0.000000	1.025163	4.422958
6	-0.000000	3.513688	2.098737
1	-0.000000	3.134748	-0.024769
1	-0.000000	3.486415	4.282485
1	-0.000000	4.598200	1.989817
6	-2.097757	0.000000	-0.747544
6	-0.000000	1.321784	-2.267970
6	2.097757	0.000000	-0.747544
6	0.000000	-1.321784	-2.267970
8	-0.000000	2.083178	-3.142812
8	3.242179	0.000000	-0.789276
8	0.000000	-2.083178	-3.142812
8	-3.242179	-0.000000	-0.789276
7	-0.000000	1.354856	1.029737
7	0.000000	-1.354856	1.029737

Table S7: TD-DFT (TDA) structure for T_3 triplet state of $[W(CO)_4(bpy)]$.

Atom Nr.	*x*	*y*	*z*
74	-0.000000	0.000000	-0.763769
6	0.000000	-0.718415	2.265618
6	0.000000	-2.698352	0.979218
6	0.000000	-1.511027	3.449048
6	-0.000000	0.718415	2.265618
6	0.000000	-3.514131	2.100918
1	0.000000	-3.133935	-0.022888
6	0.000000	-2.886894	3.370333
1	0.000000	-1.025500	4.424438
6	-0.000000	1.511027	3.449048

1	0.000000	-4.598588	1.991822
1	0.000000	-3.486343	4.283902
6	-0.000000	2.698352	0.979218
6	-0.000000	2.886894	3.370333
1	-0.000000	1.025500	4.424438
6	-0.000000	3.514131	2.100918
1	-0.000000	3.133935	-0.022888
1	-0.000000	3.486343	4.283902
1	-0.000000	4.598588	1.991822
6	-2.097410	-0.000000	-0.752014
6	-0.000000	1.320901	-2.270264
6	2.097410	0.000000	-0.752014
6	0.000000	-1.320901	-2.270264
8	-0.000000	2.082192	-3.145158
8	3.241775	0.000000	-0.796797
8	0.000000	-2.082192	-3.145158
8	-3.241775	-0.000000	-0.796797
7	-0.000000	1.355349	1.032584
7	0.000000	-1.355349	1.032584

Table S8: TD-DFT structure for T_1 triplet state of $[Mo(CO)_4(bpy)]$.

Atom Nr.	*x*	*y*	*z*
42	-0.000000	0.000000	-0.965755
6	0.000000	-0.718545	2.063205
6	0.000000	-2.697900	0.782815
6	0.000000	-1.511175	3.247849
6	-0.000000	0.718545	2.063205
6	0.000000	-3.515998	1.904620
1	0.000000	-3.142159	-0.216039
6	0.000000	-2.887045	3.172428
1	0.000000	-1.023952	4.222347
6	-0.000000	1.511175	3.247849
1	0.000000	-4.600293	1.794860
1	0.000000	-3.484507	4.087290
6	-0.000000	2.697900	0.782815

6	-0.000000	2.887045	3.172428
1	-0.000000	1.023952	4.222347
6	-0.000000	3.515998	1.904620
1	-0.000000	3.142159	-0.216039
1	-0.000000	3.484507	4.087290
1	-0.000000	4.600293	1.794860
6	-2.047149	0.000000	-0.928582
6	-0.000000	1.406121	-2.430331
6	2.047149	0.000000	-0.928582
6	0.000000	-1.406121	-2.430331
8	-0.000000	2.206635	-3.258569
8	3.194469	0.000000	-0.939802
8	0.000000	-2.206635	-3.258569
8	-3.194469	-0.000000	-0.939802
7	-0.000000	1.358498	0.834979
7	0.000000	-1.358498	0.834979

Table S9: TD-DFT (TDA) structures for T_1 triplet state of $[Mo(CO)_4(bpy)]$.

Atom Nr.	*x*	*y*	*z*
42	0.000000	-0.000000	-0.965914
6	0.000000	-0.718643	2.063212
6	0.000000	-2.698047	0.783126
6	0.000000	-1.511065	3.247985
6	0.000000	0.718643	2.063212
6	0.000000	-3.516161	1.905118
1	0.000000	-3.142338	-0.215721
6	0.000000	-2.887012	3.172672
1	0.000000	-1.023792	4.222447
6	0.000000	1.511065	3.247985
1	0.000000	-4.600465	1.795496
1	0.000000	-3.484316	4.087646
6	0.000000	2.698047	0.783126
6	0.000000	2.887012	3.172672
1	0.000000	1.023792	4.222447
6	0.000000	3.516161	1.905118

1	0.000000	3.142338	-0.215721
1	0.000000	3.484316	4.087646
1	0.000000	4.600465	1.795496
6	-2.046991	-0.000000	-0.928803
6	0.000000	1.405217	-2.431049
6	2.046991	-0.000000	-0.928803
6	0.000000	-1.405217	-2.431049
8	0.000000	2.205186	-3.259840
8	3.194347	-0.000000	-0.940390
8	0.000000	-2.205186	-3.259840
8	-3.194347	-0.000000	-0.940390
7	0.000000	1.358836	0.835268
7	0.000000	-1.358836	0.835268

Table S10: TD-DFT structures for T_3 triplet state of $[Mo(CO)_4(bpy)]$.

Atom Nr.	*x*	*y*	*z*
42	-0.000000	0.000000	-0.952788
6	0.000000	-0.718558	2.065902
6	0.000000	-2.704857	0.789632
6	0.000000	-1.507710	3.253475
6	-0.000000	0.718558	2.065902
6	0.000000	-3.517347	1.914172
1	0.000000	-3.146131	-0.210310
6	0.000000	-2.883638	3.180974
1	0.000000	-1.019304	4.227323
6	-0.000000	1.507710	3.253475
1	0.000000	-4.602275	1.809836
1	0.000000	-3.478657	4.097531
6	-0.000000	2.704857	0.789632
6	-0.000000	2.883638	3.180974
1	-0.000000	1.019304	4.227323
6	-0.000000	3.517347	1.914172
1	-0.000000	3.146131	-0.210310
1	-0.000000	3.478657	4.097531
1	-0.000000	4.602275	1.809836

6	-2.095401	-0.000000	-0.915725
6	-0.000000	1.313502	-2.452060
6	2.095401	0.000000	-0.915725
6	0.000000	-1.313502	-2.452060
8	-0.000000	2.061760	-3.334491
8	3.237983	0.000000	-0.931203
8	0.000000	-2.061760	-3.334491
8	-3.237983	-0.000000	-0.931203
7	-0.000000	1.362837	0.835646
7	0.000000	-1.362837	0.835646

Table S11: TD-DFT (TDA) structures for T_3 triplet state of [Mo(CO)$_4$(bpy)].

Atom Nr.	*x*	*y*	*z*
42	-0.000000	-0.000000	-0.954978
6	0.000000	-0.718776	2.067295
6	0.000000	-2.704315	0.791710
6	0.000000	-1.507843	3.254896
6	-0.000000	0.718776	2.067295
6	0.000000	-3.517808	1.916392
1	0.000000	-3.145425	-0.208350
6	0.000000	-2.883882	3.182249
1	0.000000	-1.019641	4.228832
6	-0.000000	1.507843	3.254896
1	0.000000	-4.602684	1.811867
1	0.000000	-3.478619	4.099033
6	-0.000000	2.704315	0.791710
6	-0.000000	2.883882	3.182249
1	-0.000000	1.019641	4.228832
6	-0.000000	3.517808	1.916392
1	-0.000000	3.145425	-0.208350
1	-0.000000	3.478619	4.099033
1	-0.000000	4.602684	1.811867
6	-2.095307	-0.000000	-0.920273
6	-0.000000	1.312540	-2.454327
6	2.095307	-0.000000	-0.920273

6	0.000000	-1.312540	-2.454327
8	-0.000000	2.060648	-3.336863
8	3.237834	-0.000000	-0.939124
8	0.000000	-2.060648	-3.336863
8	-3.237834	-0.000000	-0.939124
7	-0.000000	1.363281	0.838500
7	0.000000	-1.363281	0.838500

S2 Structure Comparisons

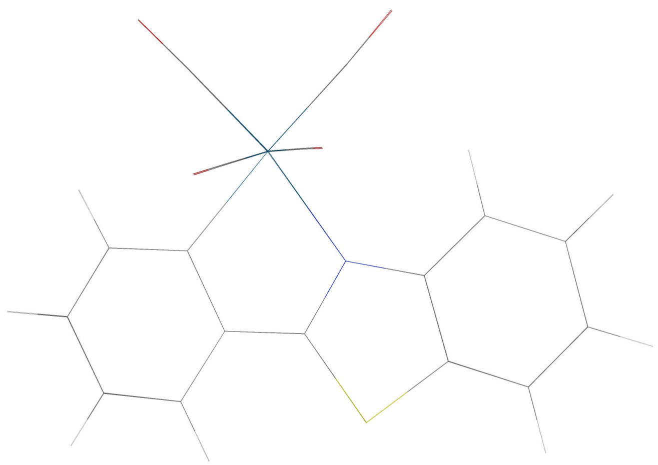

Figure S1: Overlay of TD-DFT and TD-DFT (TDA) structures for first triplet state of $[Re(CO)_4(pbt)]$ (with TDA out of plane deformation of CO ligands is slightly stronger).

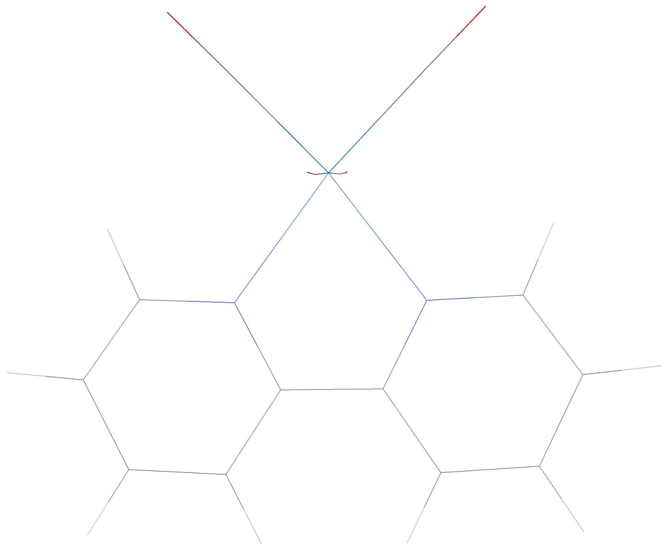

Figure S2: Identity of TD-DFT and TD-DFT (TDA) structures for first triplet state of $[W(CO)_4(bpy)]$.

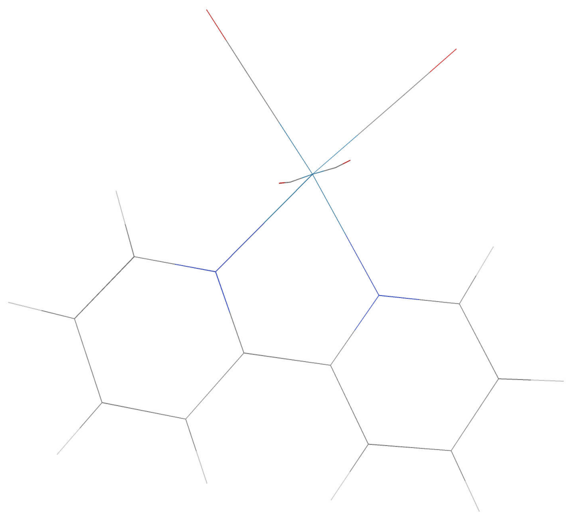

Figure S3: Overlay of TD-DFT and TD-DFT (TDA) structures for third triplet state of $[W(CO)_4(bpy)]$. The structures are virtually identical.

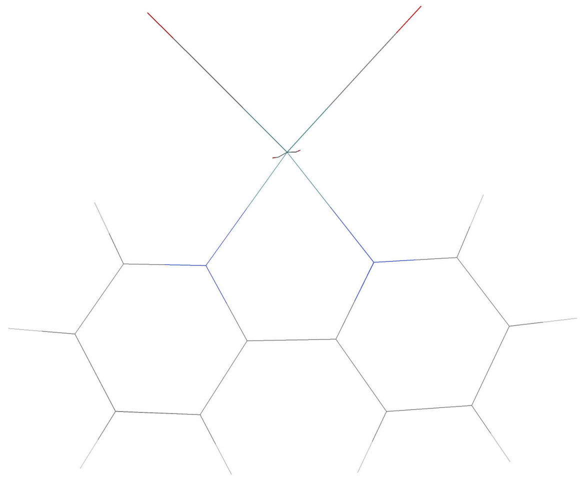

Figure S4: Overlay of TD-DFT and TD-DFT (TDA) structures for first triplet state of $[Mo(CO)_4(bpy)]$. The structures are virtually identical.

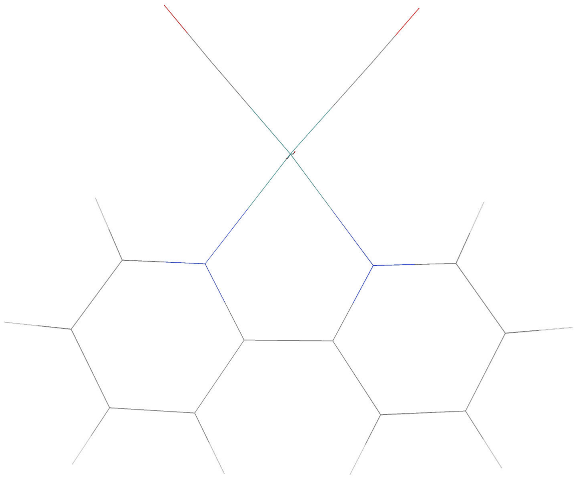

Figure S5: Overlay of TD-DFT and TD-DFT (TDA) structures for third triplet state of $[Mo(CO)_4(bpy)]$. The structures are virtually identical.

S3 Additional Calculated Spectra

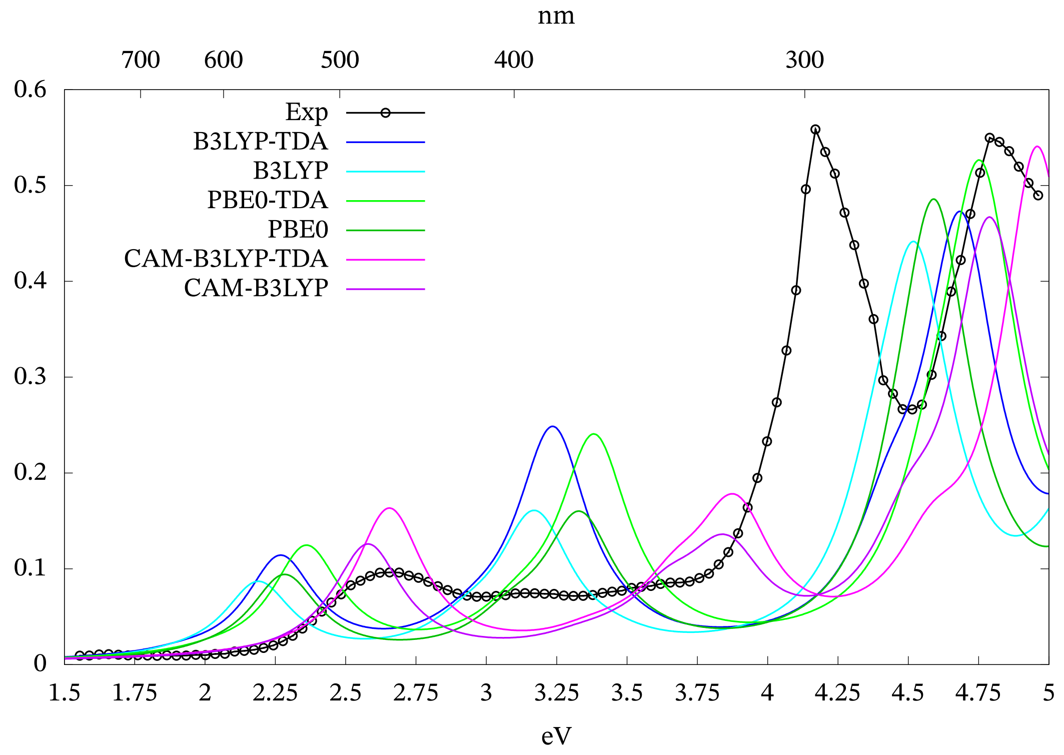

Figure S6: Calculated absorption spectrum of $[Mo(CO)_4(bpy)]$ with different denstity functionals. Experimental data presented from Reference 54.

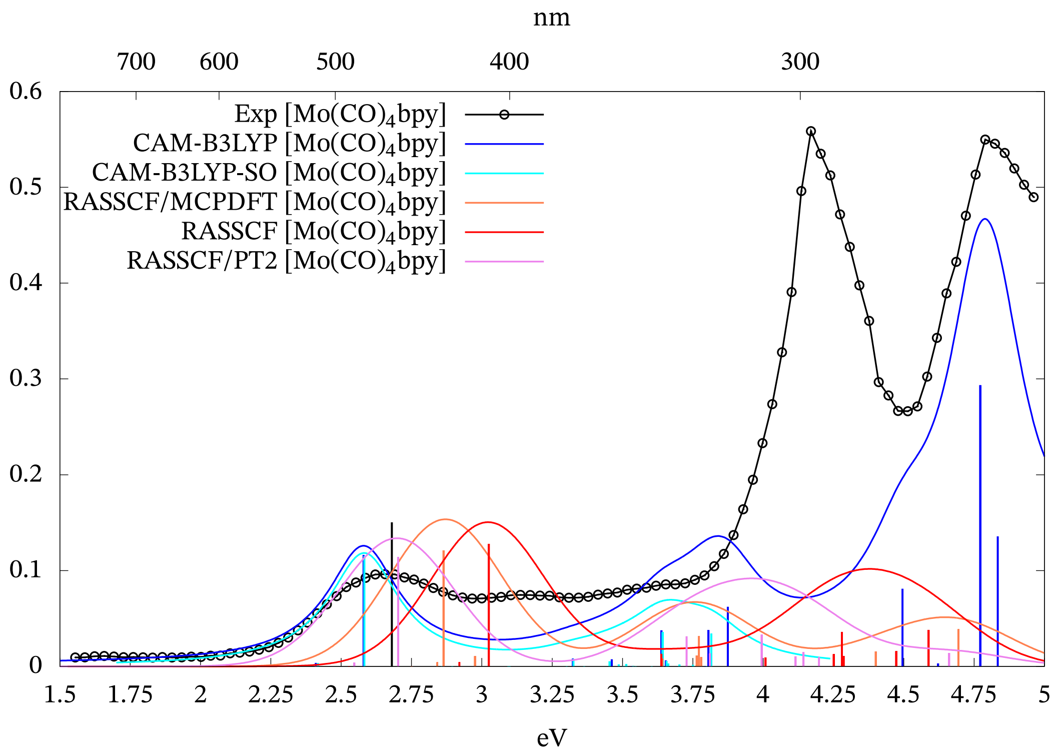

Figure S7: Calculated absorption spectrum of [$Mo(CO)_4$(bpy)] at CAM-B3LYP and multi-reference levels of theory. Experimental data presented from Reference 54.

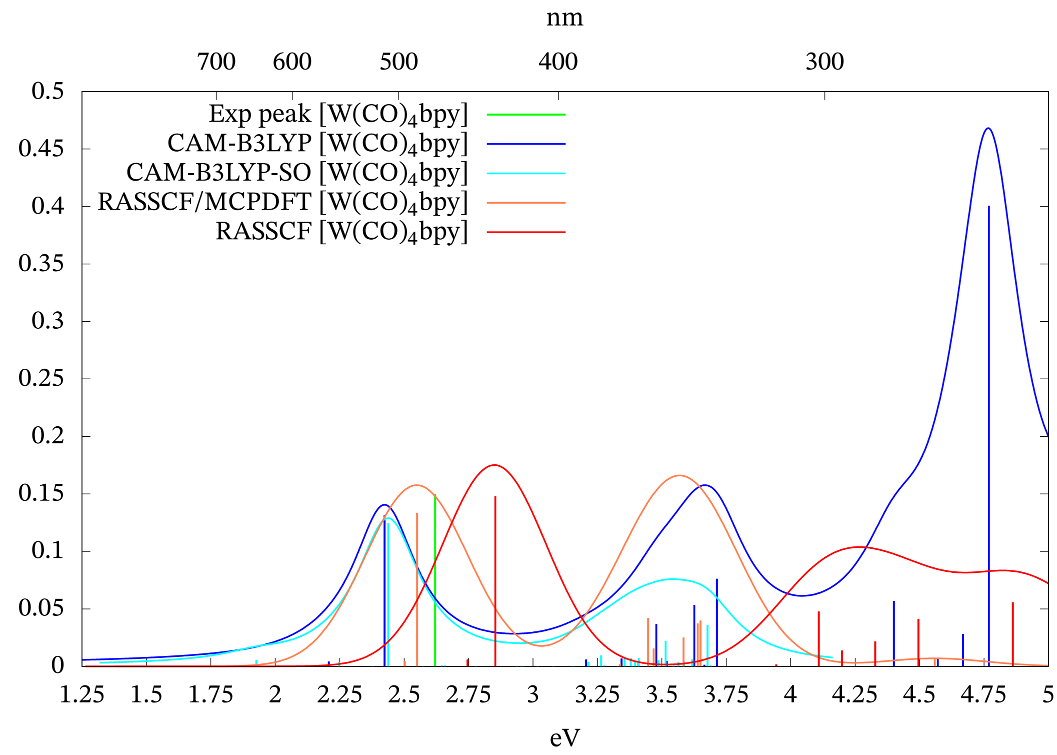

Figure S8: Calculated absorption spectrum of [$W(CO)_4$(bpy)] at CAM-B3LYP and multi-reference levels of theory. Experimental data presented from Reference 54.

S4 Electronic States

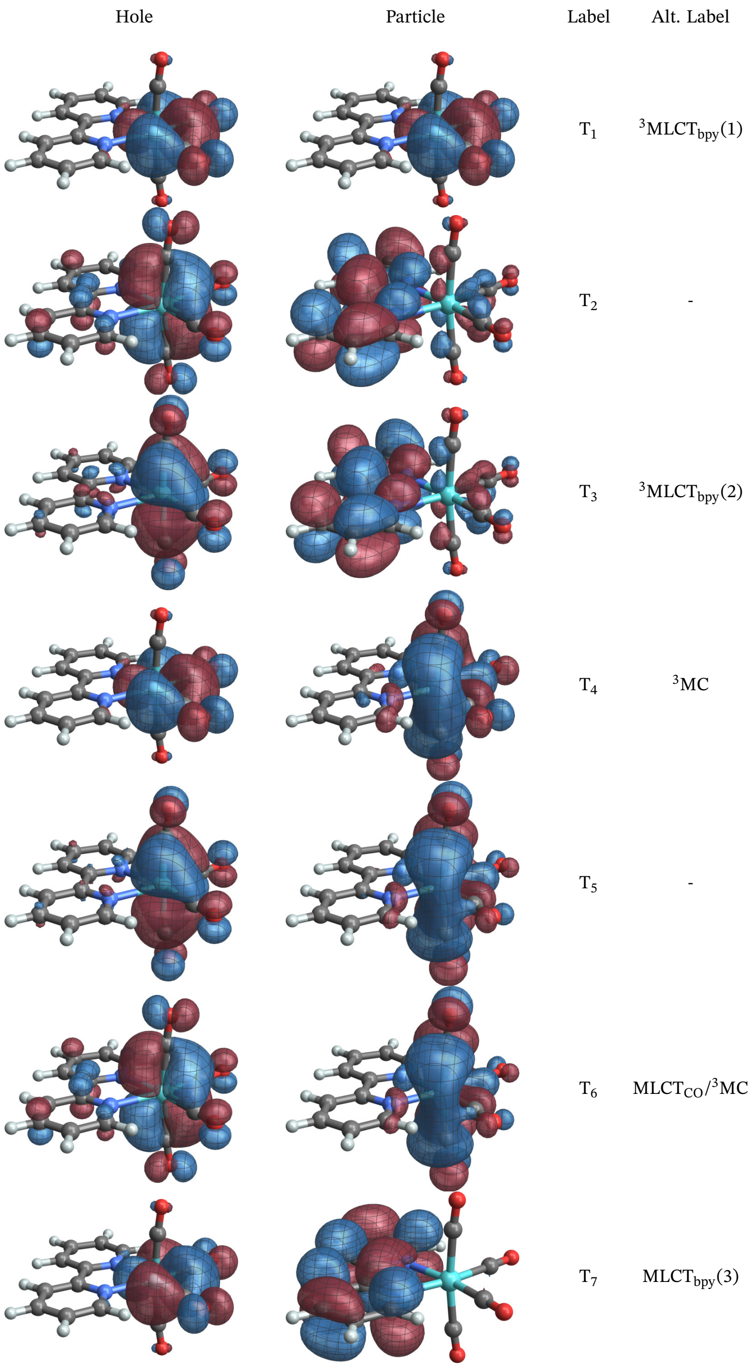

Figure S9: Excited states notation for $[Mo(CO)_4(bpy)]$. The alternative label (Alt. Label column) is according to Reference 6.

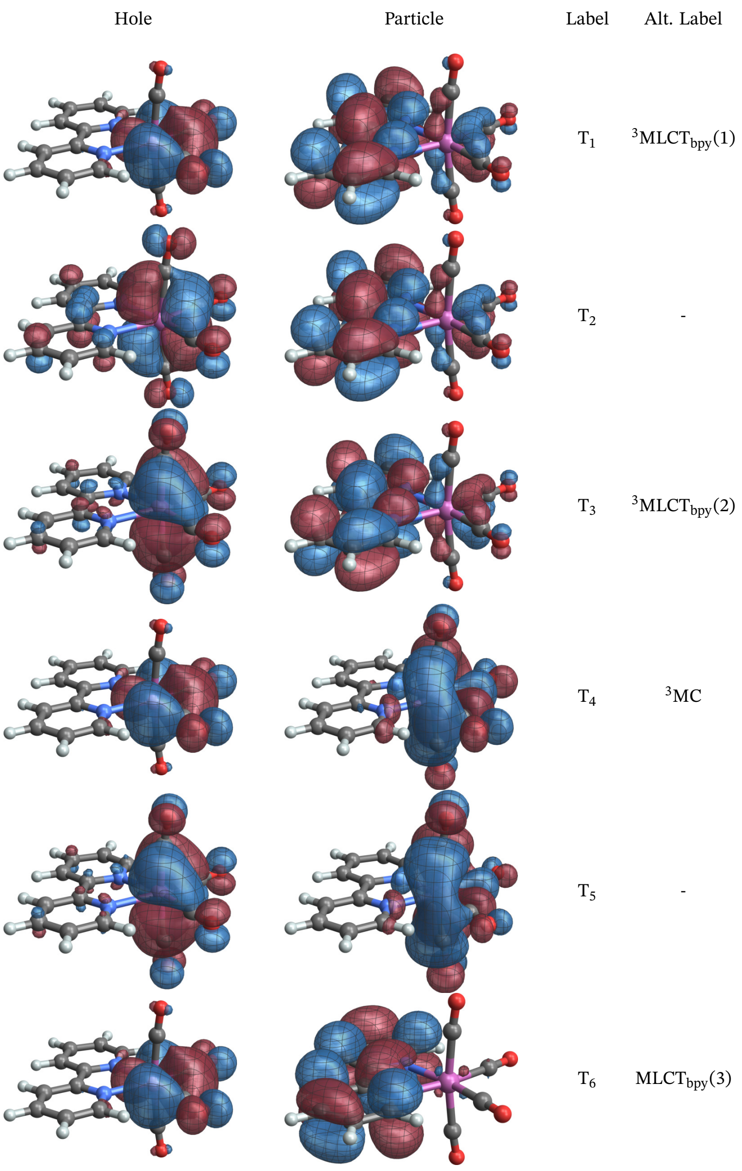

Figure S10: Excited states notation for $[W(CO)_4(bpy)]$. The alternative label (Alt. Label column) is according to Reference 6.

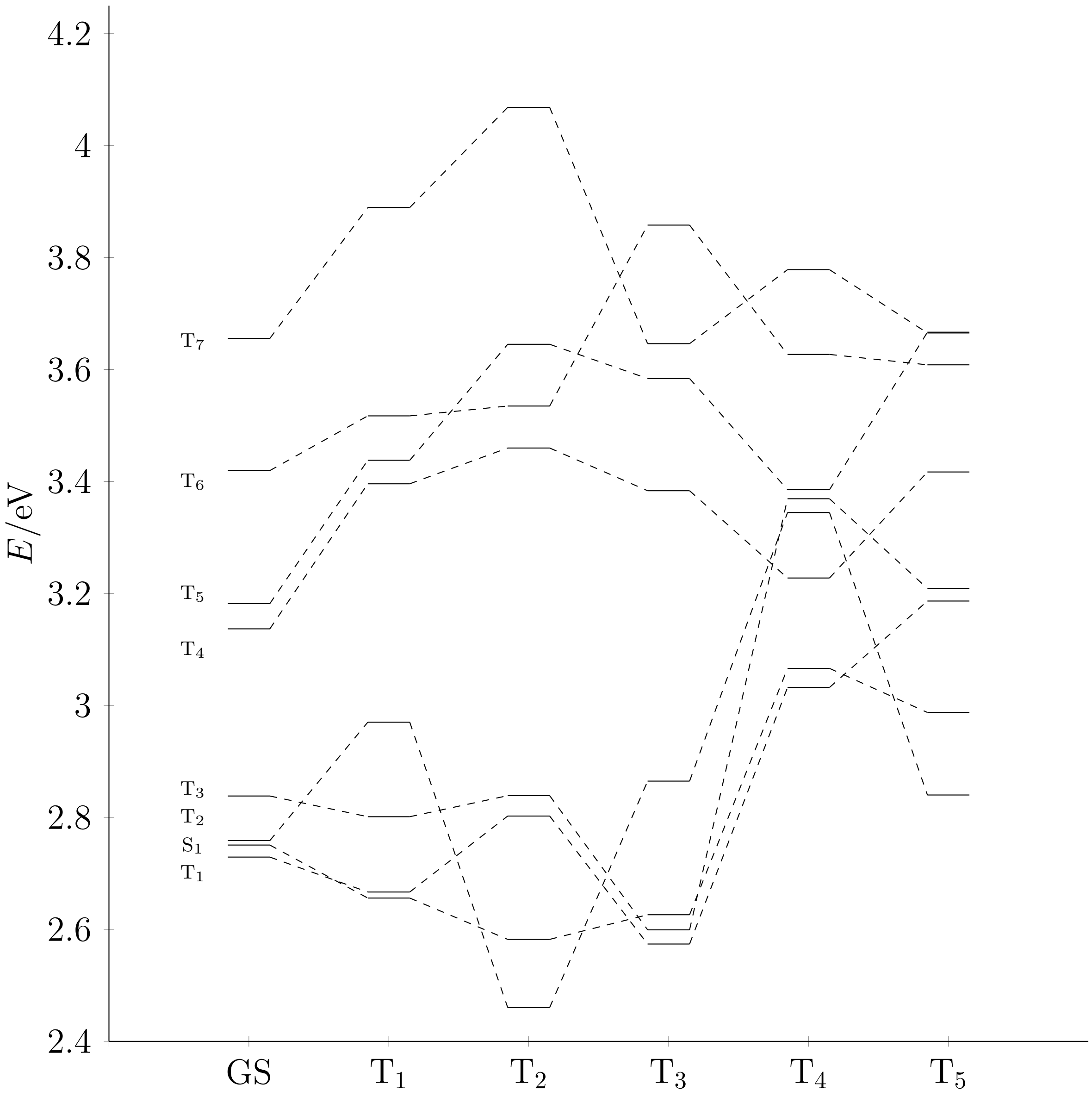

Figure S11: RASSCF(10,19)/MC-pDFT adiabatic energy diagram for first five B3LYP optimized structures [$Mo(CO)_4$(bpy)].

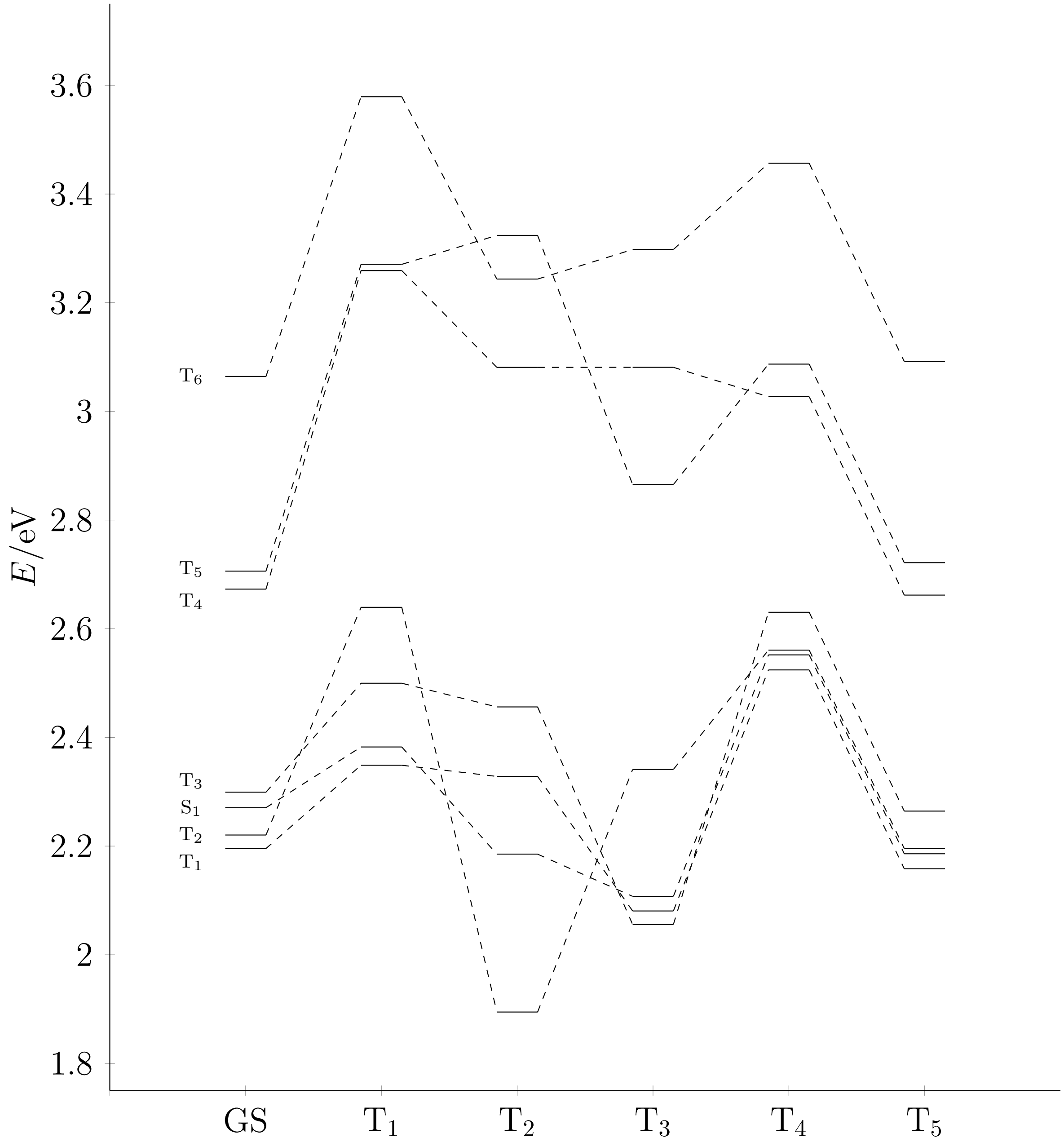

Figure S12: RASSCF(10,19)/MC-pDFT adiabatic energy diagram for first five B3LYP optimized structures [$W(CO)_4(bpy)$].

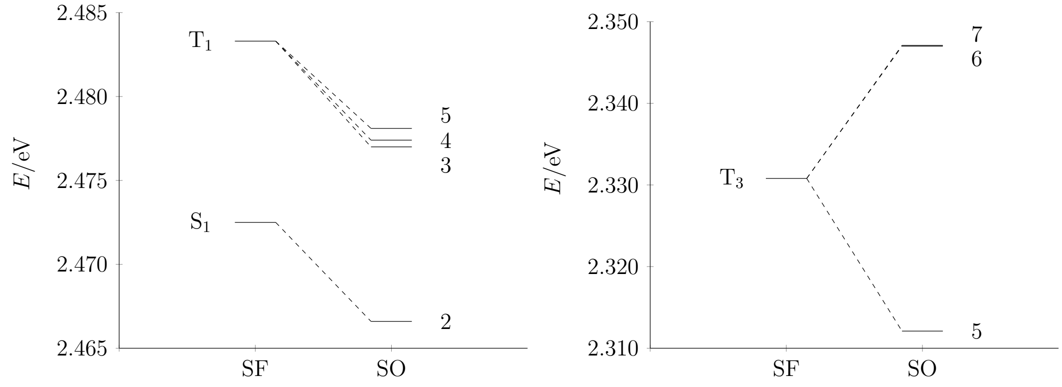

Figure S13: RASSCF/MC-pDFT levels position and split for the considered T_1 and T_3 states in $[Mo(CO)_4(bpy)]$ complex.

S5 SO-NTOs

Hole	Λ_i<br>λ_i<br>Contrib.(%)	Particle	μ_{ii}^{NTO}<br>Component x<br>Component y<br>Component z
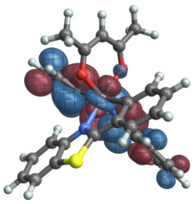	0.8583<br>$7.4 \cdot 10^{-1}$<br>98.85	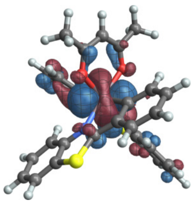	0.5206<br>−0.2921<br>−0.6169
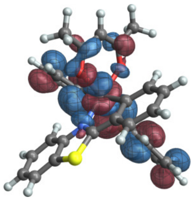	0.0742<br>$5.5 \cdot 10^{-3}$<br>0.74	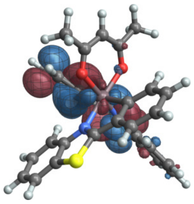	−0.1288<br>0.0475<br>0.0945
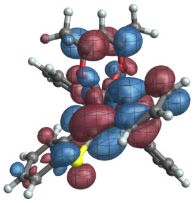	0.0441<br>$2.0 \cdot 10^{-4}$<br>0.26	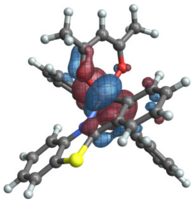	0.0282<br>0.0020<br>−0.0037
$\sum_i \lambda_i$	$7.5 \cdot 10^{-1}$		
μ_x^{tot}			0.4419
μ_y^{tot}			−0.2083
μ_z^{tot}			−0.5105

Figure S14: SO-NTOs for the 2 → 1 transition of [Ir(pbt)$_2$(acac)] at CASSCF/MC-pDFT level of theory.

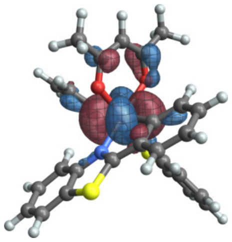

Figure S15: A linear combination of particle orbitals from SF-NTOs resembling the particle SO-NTO for the emission associated with most of the dipole intensity.

The composition of SO states becomes qualitatively different for the excited states at MC-pDFT level of theory (Figure S13). The spin-orbit states RASSCF 3 and MC-pDFT 4 contain

Hole	Λ_i<br>λ_i<br>Contrib.(%)	Particle	$\mu_{ii}^{\mathrm{NTO}}\Lambda_i$<br>Component x<br>Component y<br>Component z
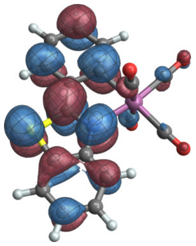	0.0232<br>$5.4 \cdot 10^{-4}$<br>98.40	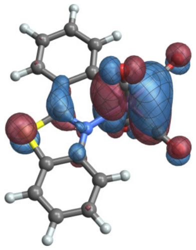	0.0273<br>0.0206<br>0.0000
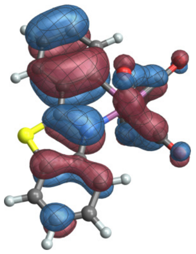	0.0026<br>$0.1 \cdot 10^{-4}$<br>1.26	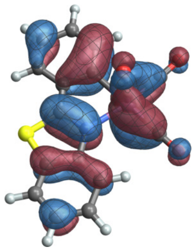	−0.0014<br>−0.0015<br>0.0000
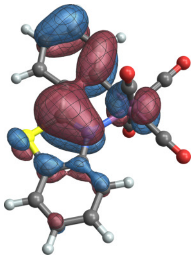	0.0010<br>$< 0.1 \cdot 10^{-4}$<br>0.17	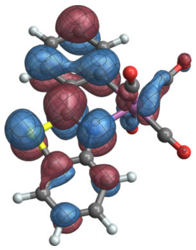	−0.0012<br>−0.0002<br>0.0000
$\sum_i \lambda_i$	$5.5 \cdot 10^{-4}$		
μ_x^{tot}			0.0250
μ_y^{tot}			0.0198
μ_z^{tot}			0.0000

Figure S16: SO-NTOs for the 3 → 1 transition of $[Re(CO)_4(pbt)]$ at the CASSCF level of theory.

the same predominant triplet components but admixture of different singlets. It is visible in different types of particle orbitals of SO-NTOs in Figures S19 and S21. SO-NTOs of the higher transition $T_3 \rightarrow S_0$ from SO states 7 (RASSCF) and 5 (MC-pDFT) are shown in Figures S20 and S22. Inconsistency in the predominated orbitals for the transition at different levels of theory is also well visible. As in previous cases it can be explained by different singlet states contributions in the spin-orbit states. Calculated transition rates with the dynamic correlation as in the tungsten complex are higher for the transition from the lowest triplet state.

Hole	Λ_i<br>λ_i<br>Contrib.(%)	Particle	$\mu_{ii}^{\mathrm{NTO}}\Lambda_i$<br>Component x<br>Component y<br>Component z
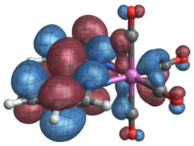	0.2011<br>$4.0 \cdot 10^{-2}$<br>96.60	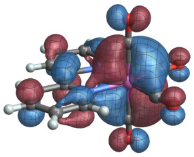	0.0000<br>−0.0000<br>−0.4439
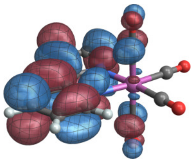	0.0249<br>$6.2 \cdot 10^{-4}$<br>1.49	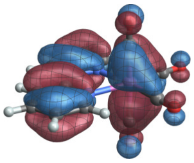	−0.0000<br>−0.0000<br>0.0348
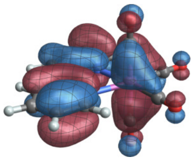	0.0160<br>$2.6 \cdot 10^{-4}$<br>0.61	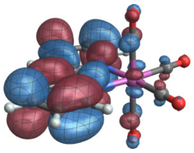	0.0000<br>−0.0000<br>−0.0048
$\sum_i \lambda_i$	$4.2 \cdot 10^{-2}$		
μ_x^{tot}			0.0000
μ_y^{tot}			−0.0000
μ_z^{tot}			−0.4083

Figure S17: SO-NTOs for the 3 → 1 transition of $[W(CO)_4(bpy)]$ at RASSCF/MC-pDFT level of theory.

Hole	Λ_i<br>λ_i<br>Contrib.(%)	Particle	$\mu_{ii}^{\mathrm{NTO}}\Lambda_i$<br>Component x<br>Component y<br>Component z
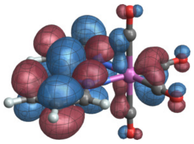	0.0405<br>$1.6 \cdot 10^{-3}$<br>85.28	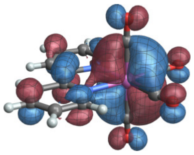	0.0000<br>−0.0000<br>0.0899
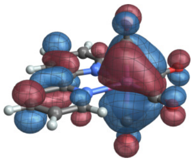	0.0125<br>$1.6 \cdot 10^{-4}$<br>8.08	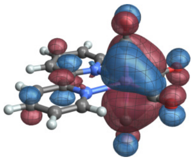	−0.0000<br>−0.0000<br>0.0133
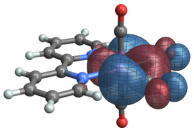	0.0103<br>$1.1 \cdot 10^{-4}$<br>5.48	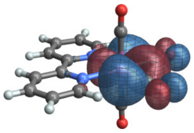	−0.0000<br>0.0000<br>−0.0267
$\sum_i \lambda_i$	$1.9 \cdot 10^{-3}$		
μ_x^{tot}			−0.0000
μ_y^{tot}			−0.0000
μ_z^{tot}			0.0698

Figure S18: SO-NTOs for the 7 → 1 transition of $[W(CO)_4(bpy)]$ at RASSCF/MC-pDFT level of theory.

Hole	Λ_i<br>λ_i<br>Contrib.(%)	Particle	$\mu_{ii}^{\mathrm{NTO}}\Lambda_i$<br>Component x<br>Component y<br>Component z
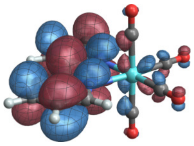	0.0687<br>$4.7 \cdot 10^{-3}$<br>98.91	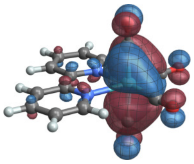	0.0000<br>−0.0243<br>−0.0000
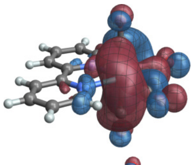	0.0064<br>$4.0 \cdot 10^{-5}$<br>0.86	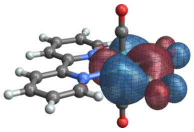	−0.0000<br>0.0094<br>−0.0000
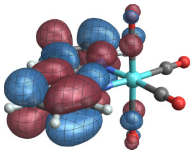	0.0016<br>$<1.0 \cdot 10^{-5}$<br>0.06	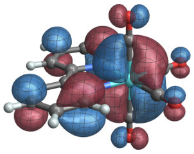	0.0000<br>−0.0038<br>−0.0000
$\sum_i \lambda_i$	$4.8 \cdot 10^{-3}$		
μ_x^{tot}			0.0000
μ_y^{tot}			−0.0149
μ_z^{tot}			−0.0000

Figure S19: SO-NTOs for the 3 → 1 transition of $[Mo(CO)_4(bpy)]$ at RASSCF level of theory.

Hole	Λ_i<br>λ_i<br>Contrib.(%)	Particle	$\mu_{ii}^{\mathrm{NTO}}\Lambda_i$<br>Component x<br>Component y<br>Component z
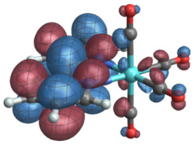	0.0397<br>$1.6 \cdot 10^{-3}$<br>97.69	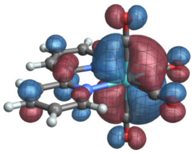	0.0000<br>0.0000<br>−0.0726
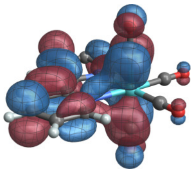	0.0046<br>$2.0 \cdot 10^{-5}$<br>1.31	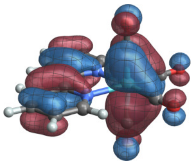	0.0000<br>−0.0000<br>0.0025
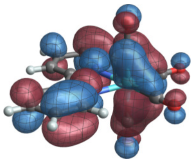	0.0027<br>$1.0 \cdot 10^{-5}$<br>0.45	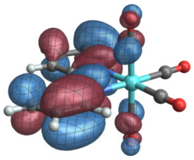	0.0000<br>−0.0000<br>−0.0066
$\sum_i \lambda_i$	$1.6 \cdot 10^{-3}$		
μ_x^{tot}			0.0000
μ_y^{tot}			−0.0000
μ_z^{tot}			−0.0653

Figure S20: SO-NTOs for the 7 → 1 transition of $[Mo(CO)_4(bpy)]$ at RASSCF level of theory.

Hole	Λ_i<br>λ_i<br>Contrib.(%)	Particle	$\mu_{ii}^{\mathrm{NTO}}\Lambda_i$<br>Component x<br>Component y<br>Component z
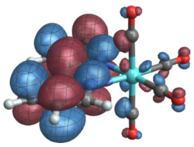	0.0846<br>$7.2 \cdot 10^{-3}$<br>98.44	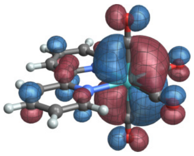	−0.0000<br>0.0000<br>0.1408
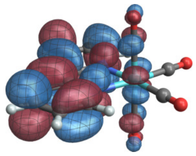	0.0074<br>$5.0 \cdot 10^{-5}$<br>0.75	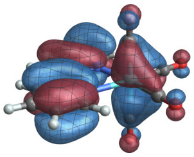	−0.0000<br>0.0000<br>−0.0067
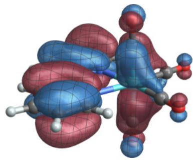	0.0045<br>$2.0 \cdot 10^{-5}$<br>0.28	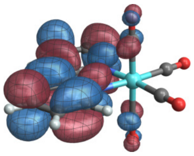	−0.0000<br>0.0000<br>−0.0028
$\sum_i \lambda_i$	$7.3 \cdot 10^{-3}$		
μ_x^{tot}			−0.0000
μ_y^{tot}			0.0000
μ_z^{tot}			0.1284

Figure S21: SO-NTOs for the 4 → 1 transition of $[Mo(CO)_4(bpy)]$ at RASSCF/MC-pDFT level of theory.

Hole	Λ_i<br>λ_i<br>Contrib.(%)	Particle	$\mu_{ii}^{\mathrm{NTO}}\Lambda_i$<br>Component x<br>Component y<br>Component z
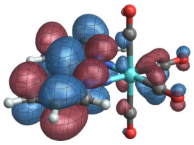	0.0293<br>$8.6 \cdot 10^{-4}$<br>97.85	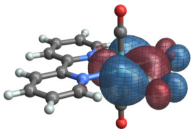	0.0000<br>0.0000<br>−0.0505
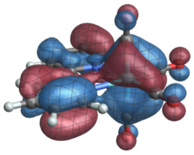	0.0032<br>$1.0 \cdot 10^{-5}$<br>1.16	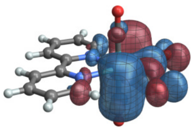	0.0000<br>−0.0000<br>0.0014
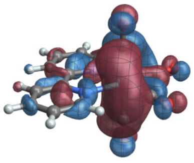	0.0019<br>$<1.0 \cdot 10^{-5}$<br>0.39	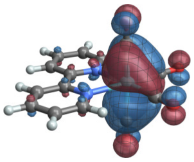	0.0000<br>−0.0000<br>−0.0045
$\sum_i \lambda_i$	$8.8 \cdot 10^{-4}$		
μ_x^{tot}			0.0000
μ_y^{tot}			−0.0000
μ_z^{tot}			−0.0432

Figure S22: SO-NTOs for the 5 → 1 transition of $[Mo(CO)_4(bpy)]$ at RASSCF/MC-pDFT level of theory.
